# Observation of stacking engineered magnetic phase transitions within moiré supercells of twisted van der Waals magnets

**DOI:** 10.1038/s41467-024-49942-2

**Published:** 2024-07-08

**Authors:** Senlei Li, Zeliang Sun, Nathan J. McLaughlin, Afsana Sharmin, Nishkarsh Agarwal, Mengqi Huang, Suk Hyun Sung, Hanyi Lu, Shaohua Yan, Hechang Lei, Robert Hovden, Hailong Wang, Hua Chen, Liuyan Zhao, Chunhui Rita Du

**Affiliations:** 1 School of Physics, Georgia Institute of Technology, Atlanta, GA 30332 USA; 2 Department of Physics, the University of Michigan, Ann Arbor, MI 48109 USA; 3 Department of Physics, University of California, San Diego, La Jolla, San Diego, CA 92093 USA; 4 Department of Physics, Colorado State University, Fort Collins, CO 80523 USA; 5 Department of Materials Science and Engineering, University of Michigan, Ann Arbor, MI 48109 USA; 6 Department of Physics, Beijing Key Laboratory of Optoelectronic Functional Materials MicroNano Devices, Renmin University of China, Beijing, 100872 China; 7 Key Laboratory of Quantum State Construction and Manipulation (Ministry of Education), Renmin University of China, Beijing, 100872 China; 8 School of Advanced Materials Discovery, Colorado State University, Fort Collins, CO 80523 USA

**Keywords:** Two-dimensional materials, Spintronics, Quantum metrology, Imaging techniques, Phase transitions and critical phenomena

## Abstract

Recent demonstrations of moiré magnetism, featuring exotic phases with noncollinear spin order in the twisted van der Waals (vdW) magnet chromium triiodide CrI_3_, have highlighted the potential of twist engineering of magnetic (vdW) materials. However, the local magnetic interactions, spin dynamics, and magnetic phase transitions within and across individual moiré supercells remain elusive. Taking advantage of a scanning single-spin magnetometry platform, here we report observation of two distinct magnetic phase transitions with separate critical temperatures within a moiré supercell of small-angle twisted double trilayer CrI_3_. By measuring temperature-dependent spin fluctuations at the coexisting ferromagnetic and antiferromagnetic regions in twisted CrI_3_, we explicitly show that the Curie temperature of the ferromagnetic state is higher than the Néel temperature of the antiferromagnetic one by ~10 K. Our mean-field calculations attribute such a spatial and thermodynamic phase separation to the stacking order modulated interlayer exchange coupling at the twisted interface of moiré superlattices.

## Abstract (web summary)

van der Waals magnets can be arranged into twisted heterostructures, with the twisting leading to the formation of new magnetic phases. Here, Li, Sun, and coauthors show via NVcentre based magnetometry small angle twisted double trilayer CrI_3_ exhibits a co-existing, hybrid magnetic phase with distinct phase transition temperatures.

## Introduction

Recently, twisted two-dimensional (2D) van der Waals (vdW) magnets have emerged as a new member in the suite of moiré quantum materials, where the spin degree of freedom is controlled through the local stacking order of moiré superlattices^1–11^. As opposed to moiré quantum electronic matter such as twisted graphene^12–15^ and transition metal dichalcogenides^14–16^ that feature enhanced electronic interactions from moiré flat bands, moiré magnets rely on twist-engineered magnetic interaction competitions to realize unconventional magnetic orders and spin excitations over moiré wavelengths, for example, in twisted vdW magnet chromium triiodide CrI_3_^1–5,7–9,11^.

Few-layer CrI_3_ shows stacking-dependent interlayer magnetic exchange coupling that is ferromagnetic (FM) for the rhombohedral stacking and antiferromagnetic (AFM) for the monoclinic stacking geometry^8,17–19^, whereas the intralayer exchange coupling is FM with a strong out-of-plane easy-axis anisotropy^8,17,18^. In twisted CrI_3_ with spatially distributed rhombohedral and monoclinic stacking geometries, the competition between uniform FM intralayer exchange coupling and FM-AFM modulated moiré interlayer exchange interaction drives the formation of a range of novel magnetic orders, such as co-existing FM and AFM states within moiré supercells in twisted bilayer and double trilayer CrI_3_^1,2^, and emergent magnetization and noncollinear spins in twisted double bilayer CrI_3_^3–5^. So far, the ongoing research on twisted CrI_3_ has mainly focused on investigating spatially modulated magnetic orders of the ground states^1–5,11^, which represents only a subset of information about moiré magnetism. The local magnetic interactions, spin dynamics, and magnetic phase transitions within and across moiré supercells await exploration and are necessary for developing a comprehensive picture of moiré magnetism.

Here, we report scanning single-spin quantum sensing^20–22^ of both static magnetization and dynamic spin fluctuations of moiré magnetism hosted by twisted double trilayer (tDT) CrI_3_ across the second-order magnetic phase transition points (*T*_c_). We show that the FM region within individual moiré supercells formed in small-twist-angle tDT CrI_3_ exhibit a higher *T*_c_ up to ~58 K in comparison with that of ~48 K for their AFM counterparts resulting in a nanoscale co-existing paramagnetic(PM)-ferromagnetic (FM) phase in an intermediate temperature regime (48 K $<$ *T* $<$ 58 K), while such a phenomenon is absent in the large-twist-angle regime. Our experimental results are well explained by a proposed mean-field theoretical model of layer-resolved magnetic phases of tDT CrI_3_ taking account of stacking engineered exchange interactions at the twisted interface. The current work highlights twist engineering as a promising tuning knob to realize local control of magnetic responses at individual stacking sites, which could contribute to a broad range of emerging 2D electronic applications^23,24^. The new insights on moiré magnetism presented in this study further highlight the potential of quantum metrology tools^20^ in exploring unconventional spin-related phenomena in correlated magnetic quantum states of matter.

## Results

**Fig. 1:**
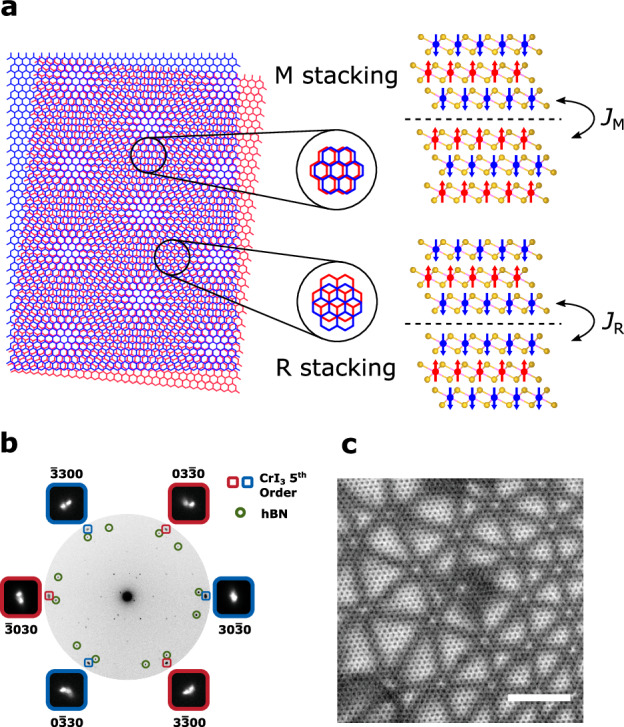
Moiré superlattices of tDT CrI_3_. **a** Left: Moiré superlattice structure of a small-twist-angle tDT CrI_3_. Only the two layers of Cr atoms adjacent to the twisted interface are shown for visual clarity. Cr atoms belonging to the top and bottom CrI_3_ layers are labeled in blue and red colors, respectively. Right: Schematic of rhombohedral (AB) and monoclinic (AB’) stacking driven FM and AFM orders in the magnetic ground state of small-twist-angle tDT CrI_3_. Magnetic moments carried by the middle two CrI_3_ monolayers are ferromagnetically or antiferromagnetically coupled depending on the local interlayer exchange interaction *J*_R_ and *J*_M_ at the twisted interface. The blue and red arrows represent the local magnetic moment carried by Cr atoms (blue and red balls) at individual layers. The yellow balls represent the I atoms and the black dashed lines highlight the twisted interface. **b** SAED patterns of the fifth-order Bragg peaks of a ~0.8° tDT CrI_3_ device (blue and red rectangles) from a surveyed sample area of ~850 nm $\times$ ~ 850 nm. **c** BF-TEM real-space image of a sample region showing the characteristic hexagonal superlattice structure in the ~0.8° tDT CrI_3_ device. The scale bar is 25 nm.

We first briefly review the pertinent material properties of tDT CrI_3_, which provides a reliably high-quality moiré magnet platform for the current study^2^. Figure 1a shows a moiré superlattice structure formed by stacking two CrI_3_ trilayers with a small twist angle. The local atomic registry exhibits a periodic modulation in real space, leading to spatially alternating stacking geometries on a length scale of moiré wavelengths^1–3^. At the monoclinic (AB’) stacking site, the two CrI_3_ trilayers are coupled by a positive exchange interaction *J*_M_, leading to local AFM order at the twisted interface with a fully compensated net magnetic moment in the ground state^2,8^. In contrast, FM order with a net magnetic moment is established at the rhombohedral (AB) stacking site driven by a negative interlayer exchange interaction *J*_R_^2,8^. Using first-principles calculations, a theoretical study has reported that the magnitude of *J*_R_ could be one order of magnitude larger than that of *J*_M_ due to the orbital-dependent exchange coupling^8^. Fundamentally, the sign and magnitude of magnetic interactions in twisted CrI_3_ not only determine its local magnetic phases but also affect their dynamic responses to external perturbations. The former has been predicted and experimentally demonstrated recently^2,8^, and the latter is the focus of the current work.

We fabricated tDT CrI_3_ devices by the standard “tear-and-stack” technique and encapsulated them with hexagonal boron nitride (hBN) nanoflakes^1–3,12,25^. The samples for transmission electron microscopy and quantum sensing measurements were fabricated separately because they required different substrates for individual measurement purposes (See Methods Section for details). Selected area electron diffraction (SAED) patterns (Fig. 1b) of a surveyed sample area of a tDT CrI_3_ device shows sets of the fifth-order Bragg peaks with sixfold rotation symmetry. The local mean twist angle is measured to be 0.8° $\pm$ 0.1° by fitting 2D Gaussians to the diffraction peaks, giving a moiré period of ~50 nm. Such an intermediate twist angle ensures a decent, regular moiré lattice structure formed in tDT CrI_3_ that is visible in transmission electron microscopy measurements. Figure 1c presents a bright-field transmission electron microscopy (BF-TEM) image, which shows the characteristic hexagonal superlattice structures with a periodicity commensurate with the moiré wavelength. The distortions from the expected moiré lattice patterns could be induced by lattice strain, relaxation, and local structural inhomogeneities (see Supplementary Information Note 1 for details)^26^.

**Fig. 2:**
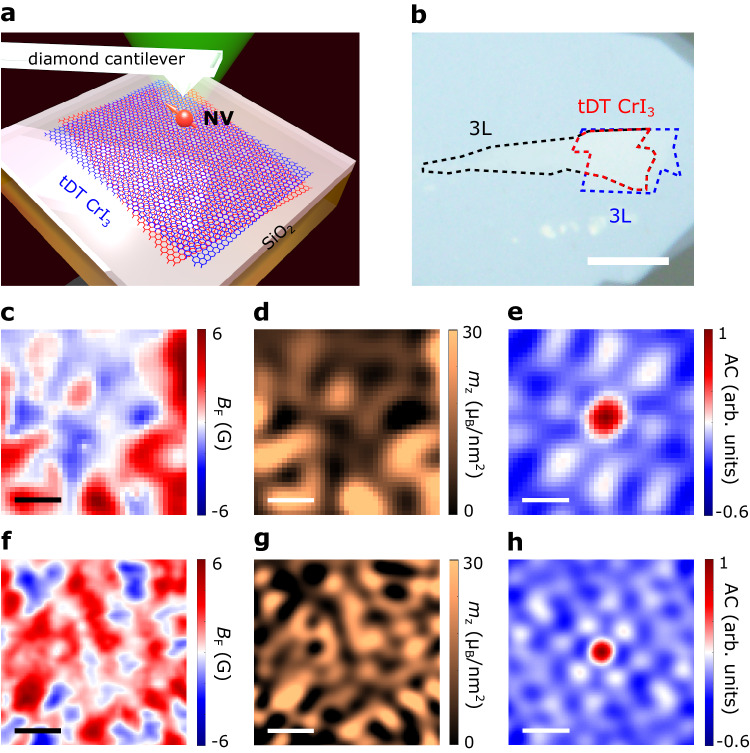
Scanning single-spin magnetometry measurements of small-twist-angle tDT CrI_3_. **a** Schematic illustration of scanning NV measurements of twisted CrI_3_. **b** Optical microscope image of a small-twist-angle tDT CrI_3_ sample. The two torn trilayer CrI_3_ flakes are outlined by the black and blue dashed lines, respectively, and the twisted area is highlighted by the red dashed lines. Scale bar is 10 μm. **c**, **f** Nanoscale scanning NV imaging of magnetic stray fields emanating from selected sample areas of a 0.15° tDT CrI_3_ (**c**) and a 0.25° tDT CrI_3_ device (**f**). **d**, **g** Magnetization maps reconstructed from the stray field patterns shown in **c** and **f** for the 0.15° tDT CrI_3_ (**d**) and 0.25° tDT CrI_3_ (**g**) sample. **e**, **h** Normalized autocorrelation (AC) maps of the stray field patterns shown in **c** and **f** for the 0.15° tDT CrI_3_ (**e**) and 0.25° tDT CrI_3_ device (**h**). Scale bar is 200 nm for images presented from **c** to **h**.

Next, we utilize scanning nitrogen-vacancy (NV) microscopy^2,18,27–29^ to spatially resolve the moiré magnetism hosted by tDT CrI_3_ as illustrated in Fig. 2a. Scanning NV magnetometry exploits the Zeeman effect to quantitatively detect local magnetic stray fields longitudinal to the NV spin axis^21^. The magnitude of the magnetic field is directly related to the splitting of NV spin energies, which can be readout by optically detected magnetic resonance measurements^2,18^ (see Supplementary Information Note 2 for details). The spatial resolution of scanning NV magnetometry is primarily determined by the NV-to-sample distance^30^, which is ~70 nm in our measurements (see Supplementary Information Note 3 for details). In the current study, we report scanning NV quantum sensing measurements of a total of three twisted vdW magnet samples: 0.15° tDT CrI_3_, 0.25° tDT CrI_3_, and 15° tDT CrI_3_. For the brevity of our narrative, 0.15° and 0.25° refer to the small-twist angle, and 15° refers to the large-twist angle in our description.

Figure 2b shows an optical microscope image of a prepared tDT CrI_3_ sample with a calibrated local twist angle $\alpha$ = 0.15° and an expected moiré period of ~250 nm. Note that in order to visualize nanoscale magnetic patterns within individual moiré supercells, here $\alpha$ is chosen to be smaller than ~0.5°, ensuring that the resulting moiré period is larger than our NV spatial sensitivity (~70 nm). Figure 2c presents a stray field *B*_F_ map measured on a selected sample area of the 0.15° tDT CrI_3_ device at 2 K. An external magnetic field of ~2000 G is applied along the NV spin axis in this measurement. The tDT CrI_3_ sample shows clear multidomain features with stray fields of opposite polarity emanating from individual domains. The reconstructed out-of-plane magnetization (*m*_z_)^2,18^ map (Fig. 2d) manifests alternating FM and AFM patches on a length scale comparable with the moiré period (see Supplementary Information Note 4 for details). The local net magnetization is measured to be 0 and ~30 μ_B_/nm^2^ for the AFM and FM domains, respectively, exhibiting the key feature of stacking-induced co-existing magnetic phases of moiré magnetism^1,2^ (see Supplementary Information Note 5 for details). The measured local magnetization of FM order in 0.15° tDT CrI_3_ also agrees with the theoretical value 29.4 μ_B_/nm^2,18^. By performing an autocorrelation operation^2^ on the magnetization map, the periodic hexagonal shaped magnetic patterns of tDT CrI_3_ is revealed in Fig. 2e, from which the local mean twist angle ($\alpha$ = 0.15°) and moiré period (~250 nm) are confirmed. The notable alternating FM-AFM state is also observed in another 0.25° tDT CrI_3_ device (Fig. 2f–h). It is worth mentioning that a larger twist angle naturally results in spatially more compact magnetic moiré patterns (Fig. 2g) with a reduced moiré period of ~150 nm (Fig. 2h).

**Fig. 3:**
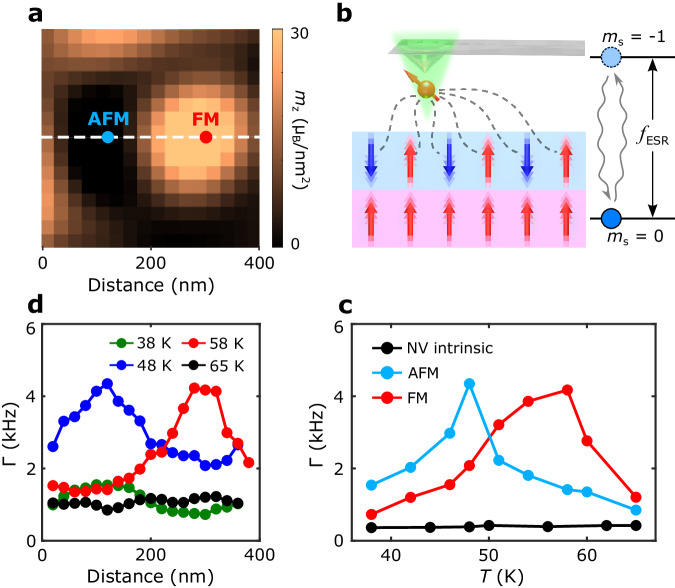
NV spin relaxometry measurements of spin fluctuations in 0.15° tDT CrI_3_. **a** Zoomed-in view of a magnetization map measured on a selected sample area (400 nm $\times$ 400 nm) of the 0.15° tDT CrI_3_ device, showing co-existing FM and AFM domains. **b** Schematic of NV spin relaxometry measurements to probe spin fluctuations of local FM and AFM states in a proximal sample. The blue and red arrows represent local magnetic moments forming spontaneous AFM and FM orders. Noncoherent magnetic noise arising from FM or AFM spin fluctuations at the NV ESR frequencies *f*_ESR_ will drive NV spin transitions from the *m*_s_ = 0 to the *m*_s_ = $\pm$1 state, resulting in enhanced NV relaxation rate. **c** Temperature dependence of NV spin relaxation rate $\Gamma$ measured when the NV center is positioned right above the FM and AFM domains formed in the 0.15° tDT CrI_3_ sample. Control measurement results are also presented to characterize the intrinsic NV spin relaxation rate. **d** 1D NV spin relaxation rate $\Gamma$ measured along the white dashed lines across the co-existing FM and AFM domains in the 0.15° tDT CrI_3_ device shown in Fig. 3a. The peak values of $\Gamma$ measured at 48 K and 58 K occur at the corresponding in-plane lateral positions of AFM and FM domains, respectively.

We now present systematic scanning NV magnetometry measurements to show distinct magnetic phase transition temperatures for the observed FM and AFM states within individual moiré supercells. Figure 3a presents a zoomed-in magnetization map of a selected sample area of the 0.15° tDT CrI_3_ device, showing two neighboring nanoscale FM and AFM domains. Due to the fully compensated net magnetic moment, temperature driven second-order magnetic phase transition of the AFM domain in tDT CrI_3_ is challenging to access by measuring its emanating magnetic flux. To circumvent this issue, here we employ NV relaxometry method^27,31–35^ to probe the intrinsic spin fluctuations at the local AFM (FM) regions. Figure 3b illustrates the mechanism of scanning NV relaxometry measurements, which takes advantage of the dipole-dipole interaction between local spin fluctuations of AFM (FM) spin density and a proximal NV center contained in a diamond cantilever. Spin fluctuations in a magnetically correlated system are driven by its time-dependent spin density distribution, for example, due to the dynamic imbalance in the thermal occupation of magnon bands with opposite chiralities^31,34,36^. For both AFM and FM systems with (un)compensated net static magnetic moment, spin-spin correlation-induced time-dependent fluctuations of the average spin density do not vanish and are expected to reach a maximum intensity around the magnetic phase transition points^33,34^. The emanating fluctuating magnetic fields at the NV electron spin resonance (ESR) frequencies will induce NV spin transitions from the *m*_s_ = 0 to *m*_s_ = $\pm$1 state, resulting in enhancement of the corresponding NV spin relaxation rates^27,32,33,36^. By measuring the spin-dependent NV photoluminescence, the occupation probabilities of NV spin states can be quantitatively obtained, allowing for extraction of NV spin relaxation rate that is proportional to the magnitude of the local fluctuating magnetic fields transverse to the NV axis (see Supplementary Information Note 6 for details)^27,32–34^. Figure 3c shows the measured temperature-dependent NV spin relaxation rate $\Gamma$ when the NV center is positioned right above the FM (AFM) domain formed in the 0.15° tDT CrI_3_ sample (Fig. 3a). Due to the divergent magnetic susceptibility, the measured NV spin relaxation rate shows a clear enhancement across the second-order magnetic phase transition points of tDT CrI_3_, from which the Curie and Néel temperatures of the co-existing FM-AFM states are measured to be ~58 K and ~48 K, respectively. We would like to highlight that similar experimental signatures are also observed in the 0.25° tDT CrI_3_ sample as detailed in Supplementary Information Note 7. It is worth noting that twist-induced lattice reconstructions for small-twist angles typically happen at the boundary of different stacking areas within individual moiré supercells in tDT CrI_3_^37^, so the potential strain effect would play a marginal role in our measurements here.

At the monoclinic (AB’) stacking sites, tDT CrI_3_ features the same type AFM interlayer interaction across the six CrI_3_ layers, thus it is not surprising that the formed local AFM moment manifests a similar *T*_c_ with that of the atomically thin pristine CrI_3_ crystals studied in previous work (see Supplementary Information Note 8 for details)^8,17,38^. In contrast, the FM order established in tDT CrI_3_ is driven by rhombohedral (AB) stacking featuring an enhanced, negative interlayer exchange coupling at the twisted interface^8,39^. Intuitively, such a stronger magnetic interaction will render the magnetic ordering more robust against thermal perturbations, resulting in an increased *T*_c_ of the local FM state formed in tDT CrI_3_. It is instructive to note that the *T*_c_ (or *T*_c_-equivalent) defined in the current manuscript for the FM order in tDT CrI_3_, an “inhomogeneous” magnetic system along the thickness direction, has considered the overall magnetic contributions from all the six CrI_3_ monolayers. Our one-dimensional (1D) scanning NV relaxometry measurements across the FM-AFM domains (Fig. 3d) further confirm this point. When *T* = 48 K, one can see that the measured 1D NV spin relaxation spectrum shows a peak value at the corresponding AFM domain site. As the temperature increases to 58 K, the observed peak of NV relaxation rate shifts to the FM domain side, demonstrating that the co-existing FM-AFM states show distinct magnetic phase transition temperatures. Note that such an experimental feature is absent in NV relaxation measurements performed at 38 K and 65 K, when the temperature is away from the Curie (Néel) points of FM (AFM) domains in 0.15° tDT CrI_3_.

**Fig. 4:**
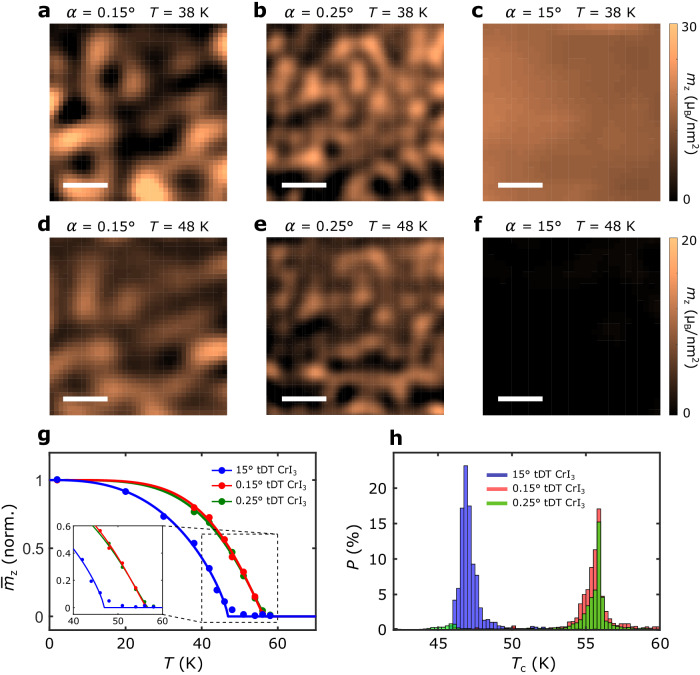
Increased Curie temperatures of FM domains formed in small-twist-angle tDT CrI_3_. **a**–**c** Reconstructed magnetization maps of selected sample areas of 0.15° tDT CrI_3_ (**a**), 0.25° tDT CrI_3_ (**b**), and 15° tDT CrI_3_ (**c**) device measured at 38 K. **d**–**f** Reconstructed magnetization maps of the same sample areas of the 0.15° tDT CrI_3_ (**d**), 0.25° tDT CrI_3_ (**e**), and 15° tDT CrI_3_ (**f**) device measured at 48 K. Scale bar is 200 nm in **a**–**f**. **g** Temperature dependence of average out-of-plane magnetization ${\bar{m}}_{{{{{{\rm{z}}}}}}}$ of FM domains formed in the selected sample areas of 0.15° tDT CrI_3_, 0.25° tDT CrI_3_, and 15° tDT CrI_3_ device. Inset shows a zoomed-in view of the magnetic curves around transition temperatures. **h** Histograms of the obtained magnetic transition temperatures of individual FM domains formed in selected sample areas of 0.15° tDT CrI_3_, 0.25° tDT CrI_3_, and 15° tDT CrI_3_ sample shown in **a**–**f**.

After showing the nanoscale stacking engineered *T*_c_ of moiré magnetism, next, we present temperature-dependent magnetization maps to provide an alternative perspective to examine the second-order magnetic phase transitions in tDT CrI_3_. Figure 4a, b presents reconstructed out-of-plane magnetization (*m*_z_) maps of the 0.15° and 0.25° tDT CrI_3_ devices measured at 38 K. It is evident that rhombohedral (AB) stacking induced FM state in tDT CrI_3_ features uncompensated net magnetization. As the temperature increases, the FM moment in tDT CrI_3_ gradually decreases and remains robust at 46 K (Fig. 4d, e). Figure 4g plots the temperature dependence of the average out-of-plane magnetization ${\bar{m}}_{{{{{{\rm{z}}}}}}}$ of FM domains formed in the selected sample areas of 0.15° and 0.25° tDT CrI_3_ devices, from which the corresponding Curie point is obtained to be ~58 K, in agreement with our NV relaxometry results. Note that in the small-twist-angle regime, local moiré lattices relax to the monoclinic (AB’) and rhombohedral (AB) stacking sites whose magnetic ground states follow their naturally preferred state. Under this condition, the interlayer exchange energy in tDT CrI_3_ is dominated by the local stacking order, and the (small) twist angle plays a secondary role. Thus, the interlayer exchange energy of 0.15° and 0.25° tDT CrI_3_ are basically the same, leading to the (almost) identical temperature-dependent magnetic phase transition behaviors. To further investigate stacking order-dependent magnetic response in tDT CrI_3_, Fig. 4c presents a magnetization map of a 15° tDT CrI_3_ device measured at 38 K. Notably, the co-existing FM-AFM phase disappears while a pure collinear FM ground state emerges in the large-twist-angle regime^1^. It is worth mentioning that the two CrI_3_ trilayers are weakly ferromagnetically coupled at the twisted interface of 15° tDT CrI_3_, resulting in a reduced Curie temperature (~48 K) in comparison with that of the small-twist-angle tDT CrI_3_. One can see that the 15° tDT CrI_3_ sample enters the paramagnetic phase showing zero net FM moment at 48 K (Fig. 4f). To better illustrate this point, Fig. 4h presents the histograms of Curie temperatures of individual FM domains formed in 0.15°, 0.25°, and 15° tDT CrI_3_ devices. Statistically, it is evident that FM domains in small-twist-angle tDT CrI_3_ show clearly higher Curie temperatures than their counterparts in large-twist-angle tDT CrI_3_ (see Supplementary Information Note 9 for details).

**Fig. 5:**
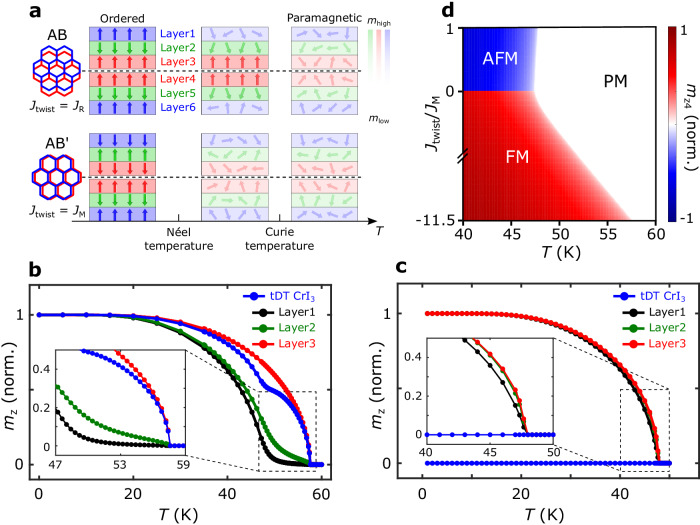
Layer-resolved magnetic phases of small-twist-angle tDT CrI_3_ with different stacking orders. **a** Schematic of layer-resolved magnetic phases of rhombohedral (AB) and monoclinic (AB’) stacked small-twist-angle tDT CrI_3_ in ordered, intermediate, and paramagnetic state. The blue, red, and green arrows represent local magnetic moment carried by individual CrI_3_ layers, and the fading background colors highlight the reduced magnetization with increasing temperature. Layers 1 to 6 are labeled from the top to bottom of tDT CrI_3_ for reference. The black dashed lines highlight the twisted interface between the two CrI_3_ trilayers. **b** Mean-field theory calculated temperature dependence of the normalized magnetization *m*_z_ of layer 1 (black), layer 2 (green), and layer 3 (red) of small-twist-angle tDT CrI_3_ with rhombohedral (AB) stacking sequence. Normalized total net magnetization *m*_z_ curve of small-twist-angle tDT CrI_3_ (blue) considering the overall contributions from the six CrI_3_ layers is also presented. Inset shows a zoomed-in view of the features around the phase transition temperatures. **c** Calculated temperature-dependent variations of the normalized magnetization *m*_z_ of layer 1 (black), layer 2 (green), and layer 3 (red) of small-twist-angle tDT CrI_3_ with monoclinic (AB’) stacking order. The total net magnetization curve of small-twist-angle tDT CrI_3_ (blue) is also presented. Inset shows a zoomed-in view around the transition temperatures. **d** A constructed mean-field phase diagram of the normalized out-of-plane magnetization of CrI_3_ layer 4 (*m*_z4_) as a function of temperature *T* and *J*_twist_/*J*_M_, highlighting the antiferromagnetic (AFM), ferromagnetic (FM), and paramagnetic (PM) states formed in small-twist-angle tDT CrI_3_.

We now present a mean-field theoretical model of atomically layer-resolved magnetic order to explain the nanoscale stacking-dependent magnetic phase transitions observed in small-twist-angle tDT CrI_3_. Our model consists of layer-uniform Ising spins representing out-of-plane magnetic moments of Cr atoms in tDT CrI_3_ as shown in Fig. 5a. The intralayer magnetic exchange interaction *J*_i_, stacking-dependent monoclinic (AB’) type interlayer exchange interaction *J*_M_, and rhombohedral (AB) type interlayer exchange interaction *J*_R_ in tDT CrI_3_ are obtained to be $-$1.32 meV, 0.086 meV, and $-$0.99 meV by comparing mean-field theory results with existing experimental transition temperatures and/or theoretically predicted values (See Method Section and Supplementary Information Note 10 for details)^40^. We compare the mean-field phases of tDT CrI_3_ with uniform monoclinic stacking (bottom panel of Fig. 5a) and rhombohedral stacking at the twisted interface (top panel of Fig. 5a), approximating the situation for regions deep inside different stacking domains. The case of monoclinic stacking features the same exchange coupling between all neighboring layers and should, therefore, show a single Néel transition at the temperature scale set by *J*_i_ and *J*_M_. However, for the rhombohedral stacking case, the much stronger *J*_R_ (in comparison with *J*_M_) between the two middle CrI_3_ layers (layer 3 and layer 4 shown in Fig. 5a) is expected to drive the system through an FM transition at a higher temperature. To corroborate the above physical picture, we have solved the mean-field equations of our model to get the temperature dependence of the normalized out-of-plane magnetization of individual CrI_3_ layers as shown in Fig. 5b, c (See Supplementary Information Note 10 for details). In the rhombohedral (AB) stacking region (Fig. 5b), one can see that the middle CrI_3_ layer (layer 3) indeed exhibits a higher *T*_c_ ~ 58 K in comparison with that of ~48 K for the monoclinic (AB’) stacking case (Fig. 5c). Moreover, in the AB stacking case the mean-field magnetic moments of the two top CrI_3_ monolayers (layer 1 and layer 2) undergo a smooth crossover as temperature decreases below *T*_c_ before reaching the saturated value but not another phase transition as dictated by the Lee-Yang theorem^41^. By considering the overall contributions from all the six CrI_3_ layers, the effective Curie temperature (*T*_c_-equivalent) of stacking induced FM order in small-twist-angle tDT CrI_3_ is calculated to be 58 K, in agreement with our NV measurement results. In contrast, in the monoclinic (AB’) stacking case (Fig. 5c), the top and middle CrI_3_ monolayers (layer 1 and layer 3 in Fig. 5a) exhibit the same temperature dependence with a *T*_c_ of ~48 K.

To further highlight the role of stacking engineering in affecting the local magnetic order and transition temperatures of small-twist-angle tDT CrI_3_, Fig. 5d plots a mean-field phase diagram of the normalized out-of-plane magnetization of CrI_3_ layer 4 (*m*_z4_) as a function of temperature *T* and *J*_twist_/*J*_M_ using our model. Here, *J*_twist_ is a variable interlayer exchange interaction at the twisted interface of tDT CrI_3_, and the sign of *m*_z4_ is defined relative to the direction of the out-of-plane magnetization *m*_z3_ of the CrI_3_ layer 3. When *J*_twist_/*J*_M_ $>$ 0, the system orders antiferromagnetically layer-wise below the *T*_c_ and *m*_z4_ is oppositely aligned with *m*_z3_ as shown in Fig. 5a. When *J*_twist_/*J*_M_ $<$ 0, the two twist-interfaced CrI_3_ monolayers are ferromagnetically coupled and uncompensated net magnetic moment is formed in tDT CrI_3_ below the magnetic critical temperature. Notably, as the magnitude of the FM-like coupling *J*_twist_ increases, the calculated *T*_c_ enhances from 48 K to 58 K when *J*_twist_/*J*_M_ reaches $-$11.5, corresponding to the rhombohedral stacking case ( *J*_twist_ = *J*_R_). In the high-temperature regime, small-twist-angle tDT CrI_3_ enters the paramagnetic phase where the long-range FM (AFM) order vanishes.

## Discussion

In summary, we have utilized scanning NV magnetometry techniques to investigate spatial and thermodynamic phase separation of moiré magnetism hosted by twisted CrI_3_. By using NV spin relaxometry methods to probe local spin fluctuations, we explicitly show that the co-existing FM-AFM phases within individual moiré supercells manifest distinct second-order magnetic phase transition points. The measured Curie temperature of the rhombohedral (AB) stacking-driven FM state is significantly higher than the Néel temperature of monoclinic (AB’) stacking-driven AFM due to the spatially modulated exchange interaction at the twisted interface. In addition, we have directly visualized the stray field and magnetization maps across the magnetic phase transition points showing that twist engineering can effectively control the Curie temperature of local FM order in tDT CrI_3_. Our results are well rationalized by a proposed mean-field theoretical model, which captures the layer-resolved *T*_c_ in small-twist-angle tDT CrI_3_. When extending to other combinations of atomically thin twisted odd number of CrI_3_ layers, we expect that the observed stacking order-dependent magnetic phase transition with separate critical temperatures remains observable by the scanning NV quantum microscopy techniques. The presented results further highlight the opportunities provided by quantum spin sensors for investigating the local spin-related phenomena in moiré quantum magnets. The stacking and temperature-driven “intracell” magnetic phase separations observed in twisted 2D magnets may also find relevant applications in realizing local control of functional material properties through carefully engineered proximity effect, advancing the current state of the art of vdW spintronic devices^24,42^.

## Methods

### Materials and device fabrications

CrI_3_ crystals used in this study were grown by the chemical vapor transport method, as reported in previous literature^3^. The tDT CrI_3_ devices are fabricated by the “tear-and-stack” method and encapsulated by hBN nanoflakes. The samples for SAED/TEM and scanning NV measurements were fabricated separately as described below, and different samples were used for SAED/TEM and NV studies. We first exfoliated the bulk crystals onto SiO_2_/Si substrates to obtain trilayer CrI_3_ and few-layer hBN. Next, the top hBN and one part of trilayer CrI_3_ were picked up by a poly(bisphenol A carbonate) stamp by sequence. The other part of trilayer CrI_3_ remained on the Si/SiO_2_ substrate and was rotated by a well-controlled angle and then picked up. The two CrI_3_ trilayer flakes were stacked with each other to form a twisted device and finally encapsulated by the bottom hBN flake. The layer number of atomically thin CrI_3_ flakes was determined by thickness-dependent optical contrast and confirmed by magnetic circular dichroism measurements^5^. The entire 2D device fabrication processes were performed inside a nitrogen-filled glovebox with oxygen level below 0.1 ppm and water level below 0.5 ppm.

For scanning NV measurements, the final hBN/tDT CrI_3_/hBN vdW stack was released onto quartz (SiO_2_) substrates with prepatterned Au striplines. The quartz substrate was chosen due to its ideal insulating nature and dielectric properties for microwave performance. The Au stripline was utilized to deliver on-chip microwave currents to realize local control of the NV spin sensor. We have prepared multiple tDT CrI_3_ samples to ensure the consistency of the presented NV results. In this paper, we reported scanning NV magnetometry studies of four CrI_3_ devices including 0.15° tDT CrI_3_, 0.25° tDT CrI_3_, 15° tDT CrI_3_, and a pristine trilayer CrI_3_ control sample (presented in Supplementary Information). Note that samples with other layer thicknesses such as small-angle twisted double bilayer CrI_3_ and twisted bilayer CrI_3_, are not studied here due to the lack of net ferromagnetism or potential device quality issues. For SAED/TEM measurements, prepared hBN/tDT CrI_3_/hBN stacks were transferred onto TEM grids with a 10-nm-thick SiN membrane. TEM images of tDT CrI_3_ with different local twist angles are presented in Supplementary Information Note 2.

### Scanning NV magnetometry measurements

The presented quantum sensing measurements were performed using a scanning NV magnetometry system consisting of a home-built confocal and a custom-designed atomic force microscope (AFM) operating in a cryo-free cryostat (attocube Inc.). A commercially available diamond cantilever (Q-Zabre LLC) containing individually addressable NV centres was glued to a quartz tuning fork for force-feedback AFM operations, and a window on top of the cryostat provided optical access for NV measurements. A sample holder with coplanar waveguides was fixed onto a stack of piezo-based positioners and scanners to engage with the diamond cantilever and perform 2D scanning measurements. We applied continuous green laser and microwave signals to carry out NV optically detected magnetic resonance measurements^36^. NV spin states were addressed by measuring NV photoluminescence using an avalanche photodiode. Microwave signals delivered to the on-chip Au stripline were supplied by a Stanford Research Systems SG386 signal generator, and the external magnetic field applied in NV measurements was generated by a three-axis superconducting vector magnet.

### NV spin relaxometry measurements

Pulsed NV spin relaxometry measurements were performed using the scanning NV magnetometry system presented above. An external magnetic field of ~1200 G was applied along the NV spin axis in these measurements, and the corresponding NV ESR frequency for the *m*_s_ = 0 to *m*_s_ = $-$1 spin transition is ~0.5 GHz. Green-laser pulses used for NV initialization and readout were generated by an electrically driven 515-nm laser. The laser power entering the objective was ~0.5 mW. The trigger pulses to the optical modulator and photon counter were generated by a programmable pulse generator, and microwave signals were modulated by a switch (Minicircuits ZASWA-2-50DR+). The top panel of Supplementary Fig. 7b shows the details of the pulsed optical and microwave sequences for our NV spin relaxometry measurements. A 1.5-μs-long green laser pulse was first applied to initialize the NV spin to the *m*_s_ = 0 state. After a delay time *t*, we measured the occupation probabilities of the NV spin at the *m*_s_ = 0 and *m*_s_ = $-$1 state by applying a microwave $\pi$ pulse on the corresponding NV ESR frequencies and measuring the spin-dependent NV photoluminescence during the first ~600 ns of the green-laser readout pulse. By measuring the integrated photoluminescence intensity as a function of the delay time *t* and fitting the data with a three-level model (See Supplementary Information Note 6 for details)^36^, NV spin relaxation rates can be quantitatively measured.

### SAED and TEM measurements

We utilized Thermo Fisher Talos, operated at 200 kV and equipped with Gatan OneView camera, to perform the SAED and TEM measurements to characterize microscopic lattice structures of tDT CrI_3_ devices encapsulated by hBN nanoflakes. The local mean twist angle of the sample was obtained by fitting 2D Gaussians to the Bragg peaks. Real-space structural image (Fig. 1c) was acquired by averaging DF-TEM images from three fifth-order Bragg peaks 120° apart. For tDT CrI_3_, the moiré domains appear √3 smaller in the TEM image(s) in comparison to the expected domain size for the corresponding twist angle $\alpha$.

### Theoretical modeling

We used mean-field theory and minimal Ising models to investigate layer-resolved magnetic phases of small-twist-angle tDT CrI_3_ with different stacking orders. Each Ising spin, representing a Cr ion, has three intralayer nearest neighbors with exchange coupling ${J}_{{{{{{\rm{i}}}}}}}$ and one nearest neighbor in an adjacent layer with interlayer exchange coupling ${J}_{{{{{{\rm{o}}}}}}}$. The Hamiltonian of the system is given by:

1 $$H=\,\mathop{\sum}_{\left\langle {jl}\right\rangle a}{J}_{{{{{{\rm{i}}}}}}}{\sigma }_{{ja}}{\sigma }_{{la}}+\mathop{\sum}_{j\left\langle {ab}\right\rangle }{J}_{{{{{{\rm{o}}}}}},{ab}}{\sigma }_{{ja}}{\sigma }_{{jb}}$$

where $j,l$ label sites within each layer and $a,b$ label layers, ${\sigma }_{{ja}}=\pm$1, ${J}_{{{{{{\rm{i}}}}}}} \, < \, 0$ (FM), and ${J}_{{{{{{\rm{o}}}}}},{ab}}$ can be negative (AB or rhombohedral stacking, denoted by ${J}_{{{{{{\rm{R}}}}}}}$ below) or positive (AB’ or monoclinic stacking, denoted by ${J}_{{{{{{\rm{M}}}}}}}$ below) depending on the stacking order. Since we do not consider magnetic ordering beyond the FM order within each layer, ${\sigma }_{{ja}}\equiv {m}_{{za}}$ is independent of $j$. We fix ${J}_{{{{{{\rm{i}}}}}}}$, ${J}_{{{{{{\rm{R}}}}}}}$, and ${J}_{{{{{{\rm{M}}}}}}}$ by requiring the mean-field ${T}_{{{{{{\rm{c}}}}}}}$ of the Ising model to be consistent with the experimental (or theoretically predicted) values, from which the following exchange couplings: ${J}_{{{{{{\rm{i}}}}}}}$ = $-$1.32 meV, ${J}_{{{{{{\rm{R}}}}}}}$ = $-$0.99 meV, and ${J}_{{{{{{\rm{M}}}}}}}$ = 0.086 meV can be obtained. To mimic the local stacking order of small-twist-angle tDT CrI_3_, we have considered different 6-layer models with AB’ stacking between adjacent layers in the top 3 (labeled by layers 1, 2, 3) and bottom 3 (labeled by layers 4, 5, 6), but either AB or AB’ stacking between layers 3 and 4. Temperature-dependent mean-field values of the layer-resolved Ising spins are solved numerically from the following nonlinear mean-field equation:

2 $$\left\langle {m}_{{za}}\right\rangle=\tanh \left[-\beta \left(3{J}_{{{{{{\rm{i}}}}}}}\left\langle {m}_{{za}}\right\rangle+\mathop{\sum}_{b}{J}_{{{{{{\rm{o}}}}}},{ab}}\left\langle {m}_{{zb}}\right\rangle \right)\right]$$

### Supplementary information

Supplementary Information

Peer Review File

## Data Availability

All data supporting the findings of this study are available from the corresponding author upon reasonable request.
