# Supplementary material for: Observation of stacking engineered magnetic phase transitions within moiré supercells of twisted van der Waals magnets
Source: Nat Commun. 2024 Jul 8;15:5712. doi: 10.1038/s41467-024-49942-2 (PMC11231268; doi:10.1038/s41467-024-49942-2)
Supplement: Supplementary file 1 — Supplementary Information [file 41467_2024_49942_MOESM1_ESM.pdf]

Supplementary Information for
Observation of stacking engineered magnetic phase transitions within moiré supercells of twisted van der Waals magnets

Senlei Li[1,+], Zeliang Sun[2,+], Nathan J. McLaughlin[3], Afsana Sharmin[4], Nishkarsh Agarwal[5], Mengqi Huang[1], Suk Hyun Sung[5], Hanyi Lu[3], Shaohua Yan[6,7], Hechang Lei[6,7], Robert Hovden[5], Hailong Wang[1], Hua Chen[4,8], Liuyan Zhao[2,*], and Chunhui Rita Du[1,3,*]

[1]School of Physics, Georgia Institute of Technology, Atlanta, Georgia 30332, USA
[2]Department of Physics, the University of Michigan, Ann Arbor, Michigan 48109, USA
[3]Department of Physics, University of California, San Diego, La Jolla, California 92093, USA
[4]Department of Physics, Colorado State University, Fort Collins, Colorado 80523, USA
[5]Department of Materials Science and Engineering, University of Michigan, Ann Arbor, Michigan 48109, USA
[6]Beijing Key Laboratory of Optoelectronic Functional Materials MicroNano Devices, Department of Physics, Renmin University of China, Beijing 100872, China
[7]Key Laboratory of Quantum State Construction and Manipulation (Ministry of Education), Renmin University of China, Beijing, 100872, China
[8]School of Advanced Materials Discovery, Colorado State University, Fort Collins, Colorado 80523, USA

[*]Corresponding authors: lyzhao@umich.edu; cdu71@gatech.edu
[+]These authors contributed equally.

Supplementary Note 1. Extended transmission electron microscopy measurements of twisted CrI_3

We use transmission electron microscopy (TEM) measurements to characterize lattice structures of prepared twisted double trilayer (tDT) CrI_3 devices at the nanoscale. Supplementary Fig. 1 presents bright-field (BF) and dark-field (DF) TEM images of a selected local sample area of a ~0.8° tDT CrI_3 device. BF-TEM image obtained from [0001] peak is equivalent to the composite DF image in domain size and shape, which shows a similar contrast. Composite DF-TEM micrographs are constructed from three fifth-order Bragg peaks ($[03\bar{3}0]$, $[\bar{3}030]$, and $[3\bar{3}00]$). Supplementary Fig. 2 shows composite DF-TEM images from different regions of prepared tDT CrI_3 devices. Characteristic hexagonal moiré superlattice structures are present over the sample areas along with local defects and lattice distortions. The local mean twist angle is calculated from domain sizes. Typically, a slightly larger twist angle will lead to a more decent and regular moiré magnetic pattern due to a reduced moiré period that is less sensitive to anisotropic lattice strain, relaxation, and local structural inhomogeneities of the CrI_3 layers as well as the uncertainty of stacking processes[1,2]. It is also instructive to note that deviations from the theoretically predicted moiré lattice structure qualitatively agrees with the spatially dependent magnetization patterns revealed by our scanning single-spin microscopy measurements.

Supplementary Note 2. Nitrogen-vacancy measurements of magnetic stray fields

Nitrogen-vacancy (NV) center is an optically active, atomic spin defect consisting of a substitutional nitrogen atom next to a carbon atom vacancy in a diamond crystal lattice[3,4]. The negatively charged NV center is a spin-triplet S = 1 system, where the $m_s = \pm 1$ spin states are degenerate at an electron spin resonance (ESR) frequency f_{ESR} of ~2.88 GHz at 2 K in absence of an external magnetic field. A static magnetic field B_{NV} longitudinal to the NV spin direction will introduce Zeeman splitting of the NV spin energies to separate the $m_s = -1$ and $m_s = +1$ spin state by an energy gap of $2\gamma B_{\mathrm{NV}}$, where γ = 2.8 MHz/G is the gyromagnetic ratio of NV centers. Supplementary Fig. 3a shows the variation of f_{ESR} as a function of B_{NV}, where $f_{\mathrm{ESR}} = 2.88 \pm 2\gamma B_{\mathrm{NV}}$ in the low field regime ($B_{\mathrm{NV}} \leq B_0$). Here, B_0 characterizes the longitudinal magnetic field that makes the NV ESR energy f_- corresponding to the lower spin transition between the $m_s = 0$ and $m_s = -1$ state to be zero. In the high field regime where $B_{\mathrm{NV}} \geq B_0$, $f_- = \gamma(B_{\mathrm{NV}} - B_0)$[5].

For NV ESR measurements presented in the current work, we apply continuous green laser to measure the fluorescence of a single NV center contained in a diamond cantilever as a function of the applied microwave frequency. Under the ESR condition, the NV spin will be excited to the $m_s = \pm 1$ states which are more likely to relax through a non-radiative pathway back to the $m_s = 0$ ground state and emit reduced photoluminescence (PL)[4]. Such spin dependent NV PL can be optically detected by an avalanche photo diode in a confocal microscope. Supplementary Fig. 3b shows a representative NV ESR spectrum measured on a tDT CrI_3 sample at 2 K. An external magnetic field B_{ext} of ~2,000 G is applied along the NV spin axis to ensure the optimal optical contrast for NV measurements. In such a large field regime, the local magnetic field B_{NV} parallel to the NV spin direction can be obtained as:

$$B_{\mathrm{NV}} = f_{\mathrm{ESR}} / \gamma + B_0 \tag{1}$$

After subtracting the contribution of B_{ext}, magnetic stray field B_F exclusively arising from the CrI_3 sample can be quantitatively obtained. By scanning the diamond cantilever over the sample surface of interest, we can map the spatially varying B_F as shown in Figs. 3c and 3f in the main text.

Supplementary Note 3. Characterizations of NV-to-sample distance

In this section, we present the detailed method to characterize NV-to-sample-distance, which determines the ultimate spatial resolution of scanning NV microscopy measurements in this work. Supplementary Fig. 4a shows a schematic of our measurement system and the coordinate system used for numerical analysis. An NV center is positioned above a pristine trilayer CrI_3 sample, which lies in the *x-y* plane. The direction $\mathbf{e}_{\mathbf{NV}}$ of the NV spin is characterized by the polar and azimuthal angles θ and φ, which are 54° and 180° in the current measurement configuration. The vertical distance d between the NV center and the top surface of the CrI_3 sample can be quantitatively measured by mapping spatial dependence of the magnetic stray field B_F arising from the CrI_3 flake. Supplementary Fig. 4b shows a set of one-dimensional (1D) (along the *x*-axis direction) B_F data measured across the edge of the CrI_3 sample. Note that the presented scanning NV measurement was performed in a "contact" mode, where the diamond cantilever is attached to the surface of the sample with a stand-off distance close to zero. Considering the dipole interaction between the NV center and magnetic sample, B_F can be expressed by the following equation[6]:

$$B_{\mathrm{F}} = \mathbf{B}^{\mathrm{edge}} \cdot \mathbf{e}_{\mathrm{NV}} \tag{2}$$

where $\mathbf{B}^{\mathbf{edge}}$ is a vector field with the *x*, *y*, *z* components to be[6]:

$$\begin{cases} B_x^{\ \mathrm{edge}}(x) = \dfrac{\mu_0 m_z d}{2\pi(x^2+h^2)} \\ B_y^{\ \mathrm{edge}}(x) = 0 \\ B_z^{\ \mathrm{edge}}(x) = -\dfrac{\mu_0 m_z x}{2\pi(x^2+h^2)} \end{cases} \tag{3}$$

Here, m_z is the saturation magnetization of the trilayer CrI_3 sample and h characterizes the variation of the sample height which can be simultaneously recorded by the atomic force microscopy during our scanning NV measurements. By fitting $B_F(x)$ to Eq. (3), the NV-to-sample distance d is extracted to be 68.0 ± 1.7 nm in current studies.

Supplementary Note 4. Reconstruction of static magnetization patterns of tDT CrI_3

In this section, we provide the technical details for reconstructing magnetization distributions of CrI_3 samples through measured stray field maps. Spatially dependent stray field $\mathbf{B}_F(\mathbf{R})$ generated by a given two-dimensional (2D) magnetic system is primarily determined by its surface magnetic moment density $\mathbf{m}(x', y')$ as follows[7]:

$$\mathbf{B}_{\mathrm{F}}(\mathbf{R}) = \int_{\Omega} dx'dy'\mathcal{D}(\mathbf{R},\mathbf{R}')\mathbf{m}(x',y'), \tag{4}$$

where $\mathcal{D}(\mathbf{R},\mathbf{R}') = -\nabla_{\mathbf{R}}\nabla_{\mathbf{R}'}\left(1/\left|\mathbf{R}-\mathbf{R}'\right|\right)$ is the magnetostatic Green's function tensor between coordinates $\mathbf{R}$ = (x, y, z) and $\mathbf{R}'$ = (x', y', z'), and Ω represents the space sample of interest. Note that the coordinate system used for numerical analysis has been presented in Supplementary Fig. 4a. In the case of atomically thin CrI_3 flakes in the current study, we have assumed a uniform magnetization distribution along the thickness direction of the sample and do not consider variations of the Green's function tensor along the sample thickness direction. In order to extract the magnetic stray field on the plane where the NV spin sensor is located (z = d), we relate the magnetic field and moment density in the Fourier space:

$$\mathbf{B}_{\mathrm{F}}(\mathbf{k},d) = D(\mathbf{k},d)\mathbf{m}(\mathbf{k}). \tag{5}$$

where $\mathbf{k} = (k_x, k_y)$ and $D(\mathbf{k}, d)$ represents the Green's function tensor in the Fourier space for $\alpha,\beta = x, y$ [7]:

$$\begin{cases} D_{\alpha\beta}(\mathbf{k},d) = -2\pi\left(k_\alpha k_\beta / k\right)e^{-kd}, \\ D_{\alpha z}(\mathbf{k},d) = -2\pi i k_\alpha e^{-kd}, \\ D_{zz}(\mathbf{k},d) = 2\pi k e^{-kd}, \end{cases} \tag{6}$$

where $k = |\mathbf{k}|$. Due to the spontaneous out-of-plane anisotropy, the local CrI_3 magnetization orients along the z-axis direction in the magnetic ground state: $\mathbf{m}(\mathbf{k}) = m_z(\mathbf{k})\hat{\mathbf{z}}$. Here we focus on the stray field along the NV spin axis direction:

$$B_{\mathrm{F}}(\mathbf{k},d) = 2\pi e^{-kd}(k\cos\theta - ik_x\sin\theta\cos\phi - ik_y\sin\theta\sin\phi)m_z(\mathbf{k}), \tag{7}$$

where θ and ϕ are the polar and azimuth angles characterizing the NV direction as shown in Supplementary Fig. 4a. Lastly, we introduce inverse Fourier transform on Eq. (7) to reconstruct the CrI_3 magnetization maps in real space. Supplementary Figs. 5a-5b present two stray field maps of a surveyed sample area of a 0.25° tDT CrI_3 device measured at 38 K and 48 K, respectively. Using the method described above, we can reconstruct the corresponding magnetization patterns of as shown in Supplementary Figs. 5c-5d.

Supplementary Note 5. Magnetization structures within individual moiré supercells of small-angle twisted CrI_3

In this section, we simulate the local magnetization structure of individual moiré cells of small-angle twisted CrI_3 using an established method reported in Refs. 5 and 8. Although the presented calculations focus on the twisted bilayer CrI_3 system, the physical picture can be readily extended to tDT CrI_3 because theoretically they should share similar magnetic moiré structures[5]. To calculate the nanoscale magnetic textures within individual moiré supercells, we start from spatially dependent interlayer stacking order established in 0.25° twisted bilayer CrI_3. Supplementary Fig. 6a shows the coordinate system used for our calculations. The total elastic and layer interaction energy E_{tot} of the moiré superlattice of twist bilayer CrI_3 can be expressed as[8]:

$$E_{\mathrm{tot}}(u_1,u_2) = E_{\mathrm{inter}}(u_1,u_2) + \sum_{i=1,2} E_{\mathrm{intra}}(\nabla u_{\mathrm{i}}) \tag{8}$$

Where u_1 and u_2 represent the local positions of lattice sites on the two CrI_3 layers (layer 1 and 2). E_{intra} and E_{inter} are the intralayer elastic energy and interlayer interaction energy of the two CrI_3 monolayers, respectively. Here, we assume that the layer 1 is fixed, and the local position u_2' of the lattice site on layer 2 after lattice relaxation can be obtained by minimizing Eq. (8) with respect to u_2. Supplementary Fig. 6b plots the relaxation induced local lattice variations of the CrI_3 layer 2: $\Delta u = u_2' - u_2$, indicating that the formed moiré structure evolves to a periodic pattern consisting of monoclinic and rhombohedral regions within a moiré supercell. Next, we calculate the local magnetic order of a moiré supercell of 0.25° twisted bilayer CrI_3. Using the theoretical model proposed in Ref. 5, the magnetic energy of this system can be expressed as:

$$E = \int d^2r\left(\sum_{\alpha,\beta,l} \frac{A_{ex}}{2}(\partial_\beta m_l^\alpha)^2 - \sum_l \frac{K}{2}(m_l^z)^2 - \sum_\alpha J m_1^\alpha m_2^\alpha\right) \quad (9)$$

where α, β are indices representing the lattice sites, l is the layer number, K = 0.096 meV/Å^2 is the magnetic anisotropy, the exchange stiffness constant A_{ex} = 15.9 meV, and J is the local interlayer exchange coupling which equals to 0.086 meV and −0.99 meV for the monoclinic and rhombohedral stacking order, respectively (See Supplementary Information Note 10 for details). Supplementary Fig. 6c presents the theoretically calculated magnetization pattern of a moiré cell of twisted bilayer CrI_3 obtained by minimizing its magnetic energy [Eq. (9)]. Note that the magnetic anisotropy is sufficiently large to spontaneously align the CrI_3 magnetization along the out-of-plane (z-axis) direction in the magnetic ground state[9,10]. One can see that the local magnetic moments of layer 1 and layer 2 are parallel and antiparallel aligned with each other in the rhombohedral and monoclinic stacking region, exhibiting the co-existing ferromagnetic (FM) and antiferromagnetic (AFM) states within individual moiré supercells.

Supplementary Note 6. NV spin relaxometry measurements of CrI_3 devices

In this section, we provide the technical details of NV spin relaxometry method to detect temperature driven second-order magnetic phase transitions of atomically thin CrI_3 samples. Our measurements utilize the dipole interaction between NV centers and local spin density of a proximal CrI_3 sample. Noncoherent magnetic stray fields at the NV ESR frequencies arising from thermal fluctuations of longitudinal spin density of CrI_3 can drive NV spin transitions between the $m_s = 0$ and $m_s = \pm 1$ states as shown in Supplementary Fig. 7a[11]. Note that the transverse spin fluctuations are gapped, and their contribution is not considered here because the minimum magnon energy of CrI_3 is well above the NV ESR frequencies in our experimentally accessible magnetic field range[12]. Due to the spin dependent PL, the transition processes between the three-level NV spin state can be optically accessed by NV relaxometry method. The top panel of Supplementary Fig. 7b shows our measurement protocol. The NV center is first initialized to the $m_s = 0$ spin state by a green laser pulse. After the delay time t, we measure the spin-dependent PL either directly ($m_s = 0$ readout) or after applying a microwave π-pulse at the NV $m_s = 0 \leftrightarrow -1$ ESR frequency ($m_s = -1$ readout). The resulting occupations of the NV spin state are readout by a second green laser pulse. The delay time dependent population of the NV spin state can be obtained by solving the following three-level model equation[13]:

$$\frac{d}{dt}\begin{pmatrix} P_0(t) \\ P_+(t) \\ P_-(t) \end{pmatrix} = \begin{pmatrix} -(\Gamma_+ + \Gamma_-) & \Gamma_+ & \Gamma_- \\ \Gamma_+ & -\Gamma_+ & 0 \\ \Gamma_- & 0 & -\Gamma_- \end{pmatrix} \begin{pmatrix} P_0(t) \\ P_+(t) \\ P_-(t) \end{pmatrix} \tag{10}$$

where P_0, P_+, and P_- are the populations of an NV spin at the $m_s = 0$, $m_s = +1$, and $m_s = -1$ states, respectively, Γ_+ and Γ_- characterize the spin relaxation rates of the $m_s = 0 \leftrightarrow +1$ and $m_s = 0 \leftrightarrow -1$ transitions. We performed NV spin relaxometry measurements at B_{ext} = 1,207 G, where the NV ESR frequencies corresponding to the $m_s = 0 \leftrightarrow -1$ and $m_s = 0 \leftrightarrow +1$ transition are 0.5 GHz and 6.26 GHz. Because the magnitude of the longitudinal spin noise decreases significantly with the increasing frequency, it is reasonable to expect that the upper NV spin relaxation rate Γ_+ is negligibly small in our measurements[11,12]. Therefore, we assume that $\Gamma_+ = 0$ and the total NV spin relaxation rate $\Gamma = \Gamma_-$ in following calculations. Considering the imperfect NV initialization in real experiments, the initial NV state is given by $P_0(0) = 1 - 2\eta$ and $P_\pm(0) = \eta$, where $\eta \ll 1$[14,15]. Under this condition, NV populations of the $m_s = 0$ (-1) states as a function of the delay time t can be written as:

$$\begin{aligned} P_0(t) &= \frac{1-\eta}{2} + \frac{1-3\eta}{2} e^{-2\Gamma t} \\ P_-(t) &= \frac{1-\eta}{2} - \frac{1-3\eta}{2} e^{-2\Gamma t} \end{aligned} \tag{11}$$

Supplementary Fig. 7b shows a set of measured time dependent NV PL spectrum corresponding to $m_s = 0$ (red) and $m_s = -1$ readout (blue). The NV spin sensor is positioned above the FM region of a 0.15° tDT CrI_3 device and the measurement temperature is 48 K. The decrease (increase) of the NV PL intensity corresponding to $m_s = 0$ (-1) readout as a function of t indicates relaxation of NV spin to a mixture of the three-level spin state. For the $m_s = 0$ readout, time dependent NV PL can be related to the spin state populations through the following equation[14]:

$$\mathrm{PL}_0 = AP_0(t) + BP_1(t) + BP_{-1}(t) + C \tag{12}$$

where A and B represent the PL generated from the bright and dark states of an NV center, and C represents the constant background. For the $m_s = -1$ readout curve, the population at the $m_s = 0$ (-1) states will be swapped after applying a microwave π-pulse at the NV $m_s = 0 \leftrightarrow -1$ ESR frequency, and the PL generated from NV center after delay time t can be expressed as:

$$\mathrm{PL}_{-1} = AP_{-1}(t) + BP_1(t) + BP_0(t) + C \tag{13}$$

Note that P_1 is a constant in Eq. (12) and Eq. (13) because $\Gamma_+ = 0$. Thus, the PL difference $\Delta\mathrm{PL} = \mathrm{PL}_0 - \mathrm{PL}_{-1}$ between the NV $m_s = 0$ and $m_s = -1$ curves can be described by the following equation[14]:

$$\Delta\mathrm{PL} = C_1 e^{-2\Gamma t} \tag{14}$$

where $C_1 = (A - B)(1 - 3\eta)$ is a constant. Supplementary Fig. 7c presents the corresponding ΔPL spectrum of the NV spin states shown in Supplementary Fig. 7b. By fitting the result to Eq. (14), NV relaxation rate Γ is extracted to be 4.35 kHz. By performing 1D scanning NV relaxometry measurements of the tDT CrI_3 sample at different temperatures, we can obtain the spatial and temperature dependent NV relaxation rates as shown in Figs. 3c-3d in the manuscript.

Supplementary Figs. 7d and 7e present a series of NV spin relaxation spectra recorded when the NV center is positioned right above the FM and AFM regions of the 0.15° tDT CrI_3 and 0.25° tDT CrI_3 devices. As the temperature increases from 38 K to 65 K, one can see that the measured NV relaxation rate Γ shows a peak value in the intermediate temperature regime, from which the individual magnetic phase transition points of the co-existing FM and AFM states of tDT CrI_3 can be obtained.

Supplementary Note 7. NV spin relaxometry measurements for 0.25° tDT CrI_3

Next, we present NV relaxometry measurements results for a 0.25° tDT CrI_3 device, showing different magnetic phase transition temperatures for FM and AFM states of moiré magnetism. Supplementary Fig. 8a shows an optical microscope image of the prepared 0.25° tDT CrI_3 device. Supplementary Fig. 8b presents a zoomed-in magnetization map of a selected twisted sample area of the device. It is evident that nanoscale FM and AFM domains formed in 0.25° tDT CrI_3 feature the local uncompensated and zero net magnetization, respectively, in agreement with the theoretical prediction[5]. We positioned the diamond cantilever containing a single NV center above the sample surface and performed NV relaxometry measurements to detect local spin fluctuations in the proximal twisted CrI_3 device. Supplementary Fig. 8c presents the temperature dependent NV spin relaxation rate Γ when the NV center is located right above the FM (AFM) domains formed in 0.25° tDT CrI_3. The measured NV spin relaxation rate shows a clear enhancement across the second-order magnetic phase transition points, from which the Curie and Néel temperature of the local FM and AFM states are measured to be ~56 K and ~48 K. We also performed 1D scanning NV relaxometry measurements across the FM-AFM domains as shown in Supplementary Fig. 8d. The obtained 1D NV spin relaxation spectrum shows peak values at the corresponding AFM (FM) domain sites when $T = 48$ (56) K. Such experimental signature is absent in NV relaxometry measurements at 38 K and 65 K, when the temperature is away from the Curie and Néel points of the corresponding FM and AFM domains formed in 0.25° tDT CrI_3. Overall, the group of NV results presented in Supplementary Fig. 8 are in agreement with that measured on the 0.15° tDT CrI_3 device reported in the main text, confirming the nanoscale stacking engineered magnetic phase transitions of moiré magnetism.

Here, we would like to further extend our discussion to briefly compare the current scanning NV results with a recent wide-field quantum sensing study of tDT CrI_3[12]. The spatial resolution of wide-field techniques is mainly determined by the optical diffraction limit (~600 nm), which is much larger than the expected moiré wavelength. Thus, it essentially detects "averaged" magnetic signals from both FM and AFM domains over arrays of moiré supercells on a mesoscopic length scale (~a few micrometers). The measured magnetic transition temperature (T_c ~ 52 K) in Ref. 12 lies in an intermediate range between the T_c and T_n of the current work, which also confirms this point. In the current study, we used state-of-the-art scanning NV quantum microscopy to resolve "intracell" FM and AFM patches within individual moiré supercells. Taking advantage of the significantly improved spatial resolution of ~70 nm, we can individually access FM and AFM moiré domains to investigate their spatial and thermodynamic phase separations, uncovering new, exciting moiré magnetism physics that cannot be revealed in the previous work.

Supplementary Note 8. Magnetic phase transitions in pristine trilayer CrI_3 and 15° tDT CrI_3 devices probed by NV spin relaxometry

In this section, we extend the demonstrated NV spin relaxometry method to pristine trilayer and large-twist-angle (15°) CrI_3 samples to examine their local magnetic phase transitions. Supplementary Fig. 9a presents the temperature dependent NV spin relaxation rate Γ measured on a pristine trilayer CrI_3 device. A peak value of 7.3 kHz is observed at ~48 K, highlighting the ferromagnet-to-paramagnet phase transition of the trilayer CrI_3[16]. It is instructive to note that the obtained Curie temperature of trilayer CrI_3 is largely the same as that for the AFM phase formed in 0.15° and 0.25° tDT CrI_3. Supplementary Fig. 9b shows the temperature dependent Γ for a 15° tDT CrI_3 device, from which the Cuire temperature is measured to be ~48 K, in agreement with our scanning NV magnetometry data presented in Fig. 4 in the main text. Our results corroborate the important role of interlayer exchange coupling in determining the magnetic transition temperatures in twisted 2D magnets. The presented studies also demonstrate the functionality of NV centers in accessing local magnetic phase diagram in the family of layered low-dimensional magnetic materials.

Supplementary Note 9. Extended scanning NV magnetometry results

In this section, we present extended scanning NV magnetometry results used to extract the corresponding magnetic phase transition temperatures of CrI_3 devices in current studies. We start with the twisted CrI_3 samples. Supplementary Fig. 10 shows the reconstructed out-of-plane magnetization m_z maps of 0.15°, 0.25°, and 15° tDT CrI_3 devices measured at 2 K, 42 K, 51 K and 56 K. The selected sample areas of individual CrI_3 devices are the same as those presented in Fig. 4 in the main text. For 0.15° and 0.25° tDT CrI_3, stacking induced FM and AFM states co-exist on a length scale that is comparable with the moiré wavelengths at low temperatures as shown in Supplementary Figs. 10a-10h. AFM domains show zero net magnetic moment over the entire temperature range of study. The rhombohedral (AB) stacking driven FM moment gradually decreases with the increasing temperature as expected, and reaches a vanishingly small value around 56 K. It is worth mentioning that a slightly larger twist angle (0.25°) results in a reduced moiré period and spatially more compact alternating FM-AFM patterns. In contrast, large-twist-angle (15°) tDT CrI_3 manifests a uniform collinear FM phase over the entire probing area as shown in Supplementary Figs. 10i-10l. The uncompensated net magnetization already reaches zero at 51 K, showing a reduced Curie temperature in comparison with that of the FM moment formed in small-twist-angle tDT CrI_3.

To further confirm the stacking dependent magnetic phase transitions, we also measured temperature dependent magnetization maps of a pristine trilayer CrI_3 device as shown in Supplementary Figs. 11a-11d. At 2 K, the trilayer CrI_3 sample shows a largely uniform, robust FM moment with a magnitude of 15 μ_B/nm^2, which is consistent with the theoretical value and experimental results reported in the previous work[17]. The FM moment gradually decays as temperature increases and vanishes at ~48 K. Supplementary Fig. 11e summarizes temperature dependence of the spatially averaged out-of-plane magnetization $\overline{m}_z$ of the trilayer CrI_3 sample. The temperature dependent magnetization curve can be fitted to the empirical expression: $M(T)/M(0)=[1-(T/T_c)^{\alpha}]^{\beta}$, where T_c = 48 K, α = 7, and β = 2. Supplementary Fig. 11f presents the histograms of obtained Curie temperatures of individual pixel in the selected sample area of the trilayer CrI_3 device. One can see that the FM magnetization hosted by trilayer CrI_3 shows a highly uniform temperature behavior with the extracted Curie temperatures largely concentrated on a narrow temperature window around 48 K over the entire probing area.

Supplementary Note 10. Theoretical model of layer-resolved magnetic phases in small-twist-angle tDT CrI_3

In this section, we present a minimal Ising mean-field model to describe the stacking-dependent magnetic phase transitions observed in small-twist-angle tDT CrI_3. We start by deriving the mean-field equation for a general quadratic spin-*S* Hamiltonian:

$$H = \sum_{i<j} \boldsymbol{S}_i \cdot \overleftrightarrow{J}_{ij} \cdot \boldsymbol{S}_j + \frac{1}{2} \sum_i \boldsymbol{S}_i \cdot \overleftrightarrow{K}_i \cdot \boldsymbol{S}_i \tag{15}$$

Here i, j run through all lattice sites where the spins are located, $\overleftrightarrow{J}_{ij}$ is a 3×3 tensor standing for exchange coupling, and $\overleftrightarrow{K}_i$ represents single-site anisotropy. Thermodynamic average of a given spin is obtained as:

$$\langle \boldsymbol{S}_j \rangle = Z^{-1} \mathrm{Tr}\left(\boldsymbol{S}_j e^{-\beta H}\right) \tag{16}$$

where Z is the partition function. The mean-field Hamiltonian is obtained by ignoring quadratic terms of fluctuation $\delta \boldsymbol{S}_j \equiv \boldsymbol{S}_j - \langle \boldsymbol{S}_j \rangle$:

$$H \approx \sum_{j \neq i} \boldsymbol{S}_i \cdot \overleftrightarrow{J}_{ij} \cdot \langle \boldsymbol{S}_j \rangle + \sum_i \boldsymbol{S}_i \cdot \overleftrightarrow{K}_i \cdot \langle \boldsymbol{S}_i \rangle - E_0\left[\{\langle \boldsymbol{S}_j \rangle\}\right] \equiv \sum_i \boldsymbol{S}_i \cdot \boldsymbol{h}_i - E_0 \tag{17}$$

Since $\boldsymbol{h}_i$, E_0 are numbers and $\boldsymbol{S}_i$ on different sites commute with each other, Eq. (16) simplifies to

$$\langle \boldsymbol{S}_i \rangle = \frac{\mathrm{Tr}\left(\boldsymbol{S}_i e^{-\beta \boldsymbol{S}_i \cdot \boldsymbol{h}_i}\right)}{\mathrm{Tr}\left(e^{-\beta \boldsymbol{S}_i \cdot \boldsymbol{h}_i}\right)} \tag{18}$$

As our goal is to figure out possible layer-resolved magnetic order of few-layer CrI_3 with different stacking sequences and their temperature dependence, instead of having an accurate spin model that can capture all macroscopic properties of these systems, we consider the following minimal Ising model with one Ising spin, representing the out-of-plane spin component of a Cr ion, per sublattice in each layer. Each spin has three intralayer nearest neighbors with exchange coupling J_i and one nearest neighbor in an adjacent layer with interlayer exchange coupling J_o. The Hamiltonian of the system is given by:

$$H = \sum_{\langle jl \rangle a} J_\mathrm{i} \sigma_{ja} \sigma_{la} + \sum_{j \langle ab \rangle} J_{\mathrm{o},ab}\, \sigma_{ja} \sigma_{jb} \tag{19}$$

where j, l label sites within each layer and a, b label layers, $\sigma_{ja} = \pm 1$, $J_\mathrm{i} < 0$ (FM), and $J_{\mathrm{o},ab}$ can be negative (AB or rhombohedral stacking, denoted by J_R below) or positive (AB' or monoclinic stacking, denoted by J_M below) depending on the stacking order. Since we do not consider magnetic ordering beyond the FM order within each layer, $\sigma_{ja} \equiv m_{za}$ is independent of j. The mean-field equation Eq. (18) then becomes:

$$\langle m_{za} \rangle = \tanh(-\beta \langle m_{za} \rangle h_a) = \tanh\left[-\beta\left(3J_\mathrm{i} \langle m_{za} \rangle + \sum_b J_{\mathrm{o},ab} \langle m_{zb} \rangle\right)\right] \tag{20}$$

At the transition temperature where $\langle m_{za} \rangle \approx 0$, we can linearize Eq. (20) to get:

$$\langle m_{za} \rangle = -\beta_c\left(3J_\mathrm{i} \langle m_{za} \rangle + \sum_b J_{\mathrm{o},ab} \langle m_{zb} \rangle\right) \tag{21}$$

And the transition temperature $T_c = \frac{1}{k_B \beta_c}$ can be obtained from the largest eigenvalues of the above linear problem. We fix J_i, J_R, and J_M by requiring the mean-field T_c of the Ising model to be consistent with experimental (theoretical) values. Specifically, Eq. (21) leads to the following simplified equations for a few systems whose T_c have been measured experimentally or theoretically predicted:

$$T_c(\text{monolayer}) = \frac{|3J_i|}{k_B}$$

$$T_c(\text{few-layer, AB'}) = \frac{|3J_i - 2J_M|}{k_B} \quad (22)$$

where k_B is the Boltzmann constant, T_c(monolayer) and T_c(few-layer, AB') are the Curie (Néel) temperatures of monolayer CrI_3 and few-layer CrI_3, respectively. J_i is directly determined by the monolayer CrI_3, whose difference from that of the few-layer CrI_3 gives J_M. Using the T_c(monolayer) = 46 K[18], T_c(few-layer, AB') = 48 K (reported in the current work), J_i and J_M are calculated to be −1.32 meV and 0.086 meV, respectively. Note that the obtained J_i is close to previous literature results[19] and that estimated from the spin-flip transition field of bilayer CrI_3 (~0.7 T). Lastly, J_R = −0.99 meV is chosen so that the mean-field obtained T_c for both bulk CrI_3 and rhombohedral (AB) stacked 3L + 3L CrI_3 are in agreement with experimental results[20]. The final exchange coupling parameters in our calculations for the present small-twist-angle tDT CrI_3 system are:

$$J_i = -1.32 \text{ meV}, J_R = -0.99 \text{ meV}, J_M = 0.086 \text{ meV} \quad (23)$$

The above exchange parameters are determined in a way that not only able to reproduce the experimental transition temperatures for the tDT CrI_3 systems but are also in agreement with (not far off from) existing literature results. To mimic the local stacking order of 3L + 3L CrI_3, we have considered different 6-layer models with AB' stacking between adjacent layers in the top 3 (layers 1, 2, 3) and bottom 3 (layers 4, 5, 6), but either AB or AB' stacking between layers 3 and 4. We then solved Eq. (20) iteratively for $\langle m_{za} \rangle (T)$. Data in Figs. 5b and 5c in the main text were obtained using $J_{o,34} = J_R$ and J_M, respectively, with the other $J_{o,ab} = J_M$.

For readers' reference, if using the previously reported experimental T_c (45 K for monolayer CrI_3[10], 46 K for few-layer AB' CrI_3[10], and 61 K for bulk CrI_3[9]), the obtained (J_i, J_M, J_R) are (−1.29 meV, 0.043 meV, −0.689 meV). Using these values, the T_c of the FM state in small-twist-angle tDT CrI_3 is ~53 K and the T_N for the AFM state is ~46 K. Separately, with T_c(monolayer) = 45 K and using root-finding to get J_M and J_R so that the experimental T_c and T_N of small-twist-angle tDT CrI_3 are exactly reproduced, we have (J_i, J_M, J_R) = (−1.29 meV, 0.143 meV, −1.10 meV). Regardless of using the sets of these parameters, the layer-resolved spin order and trends of its temperature dependence reported in the main text are qualitatively unaffected. We understand that mean-field theories generally overestimate T_c, the exchange parameters obtained in this manner are expected to be smaller in magnitude than the values in a more complete microscopic model of CrI_3. Also, such an approach may oversimplify any surface-induced modifications on the spin Hamiltonian and thickness-dependent quantum and thermal fluctuations that become more pronounced in few-layer CrI_3. A more in-depth understanding of layered dependent magnetic phase transitions in tDT CrI_3 might call for further comprehensive theoretical work. We hope that our results presented here serve the purpose of stimulating future exciting studies on this topic.

References

1. Xie, H. *et al.* Twist engineering of the two-dimensional magnetism in double bilayer chromium triiodide homostructures. *Nat. Phys.* **18**, 30–36 (2022).
2. Sung, S. H. *et al.* Torsional periodic lattice distortions and diffraction of twisted 2D materials. *Nat. Commun.* **13**, 7826 (2022).
3. Doherty, M. W. *et al.* The nitrogen-vacancy colour centre in diamond. *Phys. Rep.* **528**, 1–45 (2013).
4. Rondin, L. *et al.* Magnetometry with nitrogen-vacancy defects in diamond. *Rep. Prog. Phys.* **77**, 056503 (2014).
5. Song, T. *et al.* Direct visualization of magnetic domains and moiré magnetism in twisted 2D magnets. *Science* **374**, 1140–1144 (2021).
6. Tetienne, J.-P. *et al.* The nature of domain walls in ultrathin ferromagnets revealed by scanning nanomagnetometry. *Nat. Commun.* **6**, 6733 (2015).
7. Guslienko, K. Y. & Slavin, A. N. Magnetostatic Green's functions for the description of spin waves in finite rectangular magnetic dots and stripes. *J. Magn. Magn. Mater.* **323**, 2418–2424 (2011).
8. Carr, S. *et al.* Relaxation and domain formation in incommensurate two-dimensional heterostructures. *Phys. Rev. B* **98**, 224102 (2018).
9. McGuire, M. A., Dixit, H., Cooper, V. R. & Sales, B. C. Coupling of crystal structure and magnetism in the layered, ferromagnetic insulator CrI_3. *Chem. Mater.* **27**, 612–620 (2015).
10. Kim, H. H. *et al.* Evolution of interlayer and intralayer magnetism in three atomically thin chromium trihalides. *Proc. Natl. Acad. Sci.* **116**, 11131–11136 (2019).
11. McLaughlin, N. J. *et al.* Quantum imaging of magnetic phase transitions and spin fluctuations in intrinsic magnetic topological nanoflakes. *Nano. Lett.* **22**, 5810–5817 (2022).
12. Huang, M. *et al.* Revealing intrinsic domains and fluctuations of moiré magnetism by a wide-field quantum microscope. *Nat. Commun.* **14**, 5259 (2023).
13. Du, C. *et al.* Control and local measurement of the spin chemical potential in a magnetic insulator. *Science* **357**, 195–198 (2017).
14. Ariyaratne, A., Bluvstein, D., Myers, B. A. & Jayich, A. C. B. Nanoscale electrical conductivity imaging using a nitrogen-vacancy center in diamond. *Nat. Commun.* **9**, 2406 (2018).
15. Shields, B. J., Unterreithmeier, Q. P., de Leon, N. P., Park, H. & Lukin, M. D. Efficient readout of a single spin state in diamond via spin-to-charge conversion. *Phys. Rev. Lett.* **114**, 136402 (2015).
16. Huang, B. *et al.* Layer-dependent ferromagnetism in a van der Waals crystal down to the monolayer limit. *Nature* **546**, 270–273 (2017).
17. Thiel, L. *et al.* Probing magnetism in 2D materials at the nanoscale with single-spin microscopy. *Science* **364**, 973–976 (2019).
18. Safi, A. L. & Ahmed, Md. A. CrI_3 monolayer: magnetic and electronic behaviors under biaxial strain and external electric field—a first principle study. *Eur. Phys. J. B* **95**, 57 (2022).
19. Sivadas, N., Okamoto, S., Xu, X., Fennie, Craig. J. & Xiao, D. Stacking-dependent magnetism in bilayer CrI_3. *Nano. Lett.* **18**, 7658–7664 (2018).
20. Dillon, J. F., Jr. & Olson, C. E. Magnetization, Resonance, and optical properties of the ferromagnet CrI_3. *J. Appl. Phys.* **36**, 1259–1260 (1965).

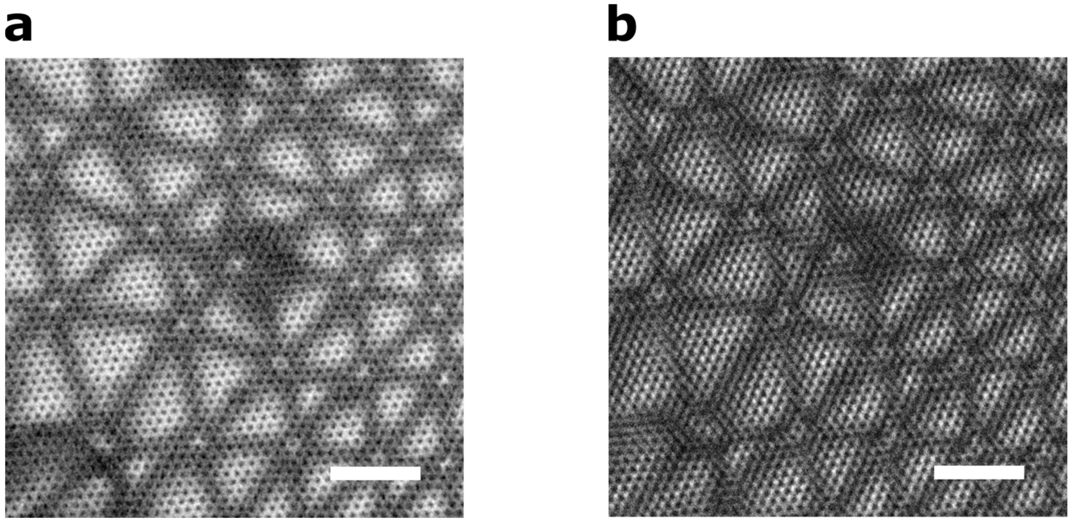

Supplementary Fig. 1. Correlated Bright Field (BF) and Dark Field (DF) composite images. **a** Contrast inverted BF-TEM image of tDT CrI_3 sample from $[0001]$ Bragg peak. **b** Composite image constructed using DF-TEM from $[3\bar{3}00]$, $[\bar{3}030]$, and $[0\bar{3}30]$ Bragg peaks. Both images exhibit the same moiré features and periodicities. The scale bar is 20 nm.

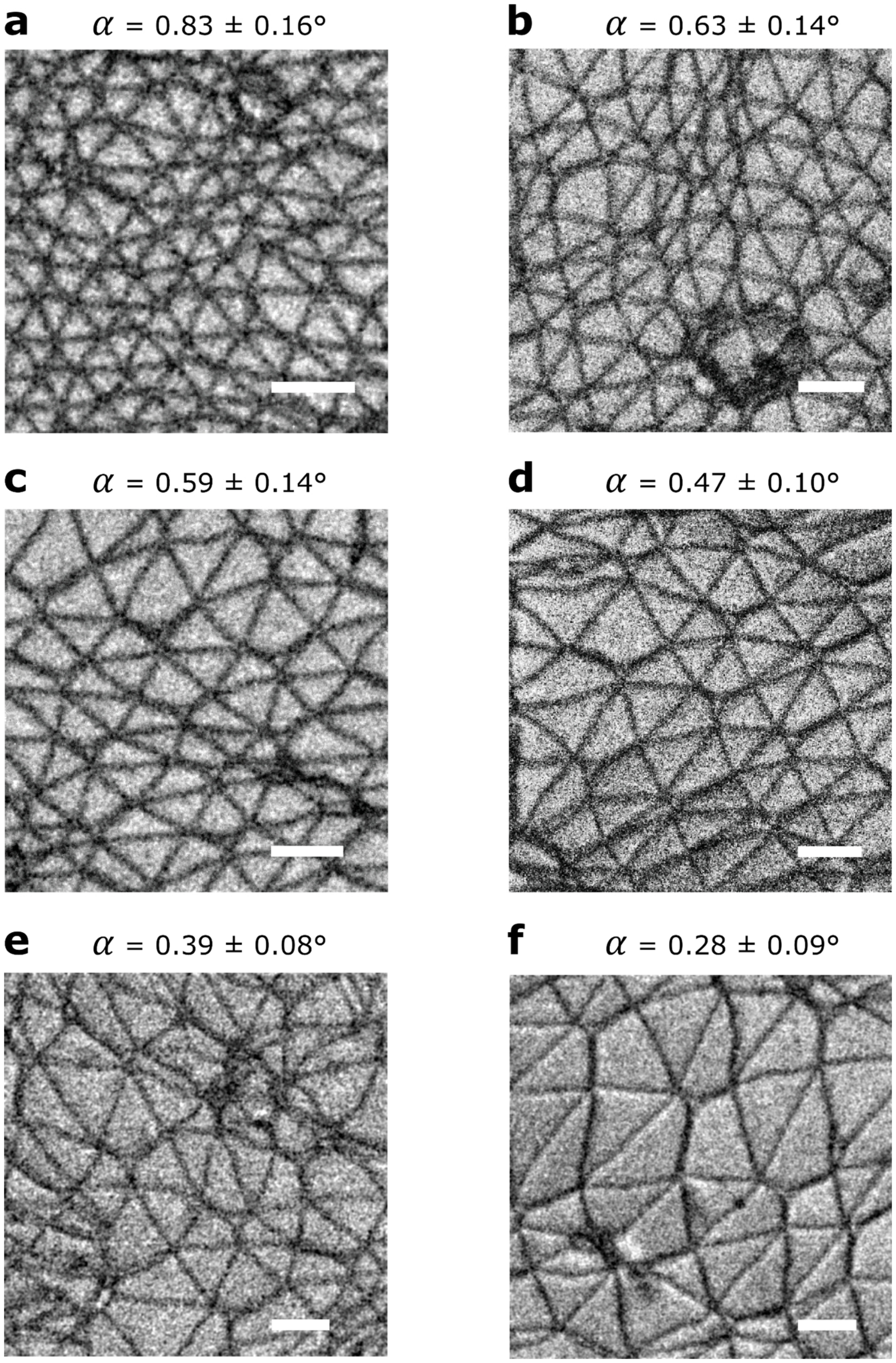

Supplementary Fig. 2. Composite DF-TEM images of tDT CrI_3. a–f Composite DF-TEM images of six different sample regions of prepared tDT CrI_3 devices. α denotes local mean twist angle. The scale bar is 50 nm.

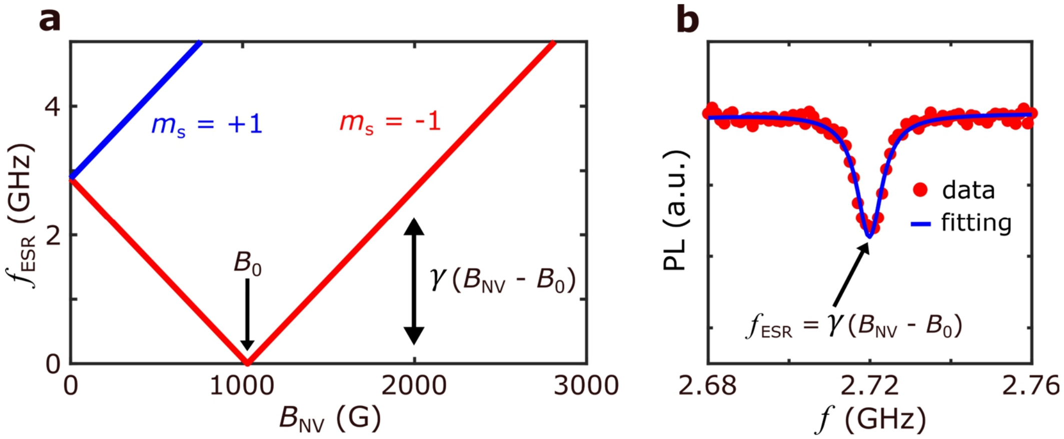

Supplementary Fig. 3. NV ESR measurements. a NV ESR frequency of $m_s = \pm 1$ states as a function of magnetic field B_{NV} along the NV spin axis. **b** A typical set of NV optically detected magnetic resonance (ODMR) spectrum measured on a tDT CrI_3 device at 2 K. An external static magnetic field B_{ext} of 2,000 G is applied along the NV spin direction.

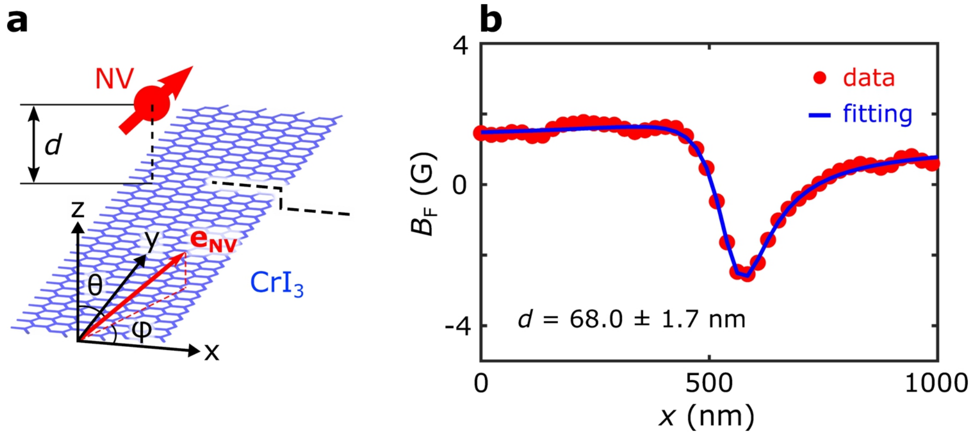

Supplementary Fig. 4. Characterization of NV-to-sample distance. a Schematic of the NV measurement platform and coordinate system for numerical analysis. **b** 1D stray field B_F spectrum measured across the edge (along the black dashed lines shown in Supplementary Fig. 4a) of a trilayer CrI_3 sample. The NV-to-sample distance d can be quantitatively obtained by fitting the spatial distribution of B_F to Eq. (3) in Supplementary Information.

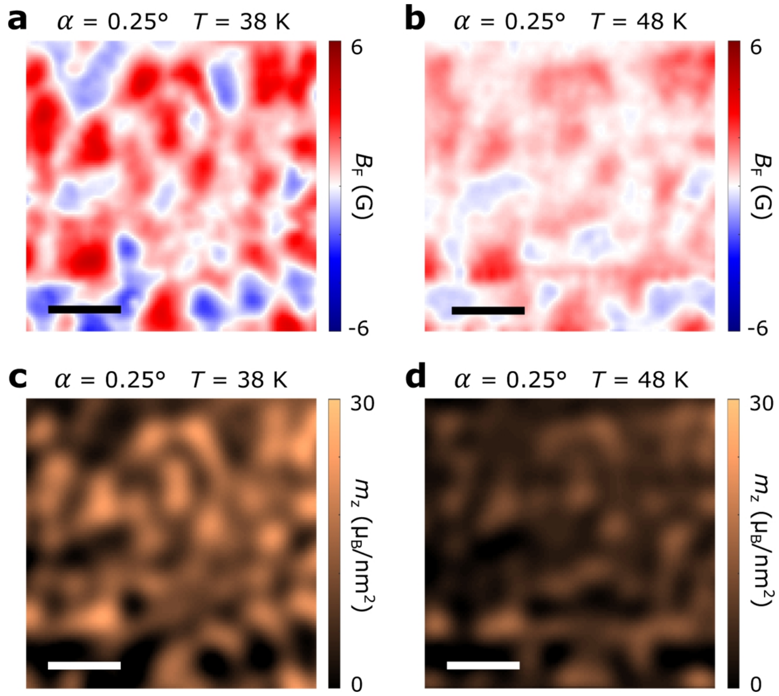

Supplementary Fig. 5. Magnetization reconstruction of tDT CrI_3. a-b Stray field maps of a surveyed sample area of a 0.25° tDT CrI_3 device measured at 38 K (**a**) and 48 K (**b**). An external magnetic field B_{ext} of 2,000 G is applied along the NV axis in these measurements. **c-d** Magnetization patterns reconstructed from the stray field maps presented in **a** and **b** at the corresponding measurement temperatures. The scale bar is 200 nm.

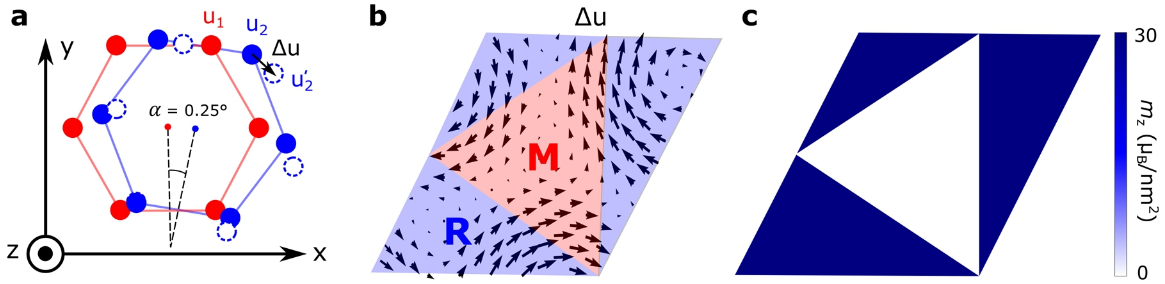

Supplementary Fig. 6. Microscopic magnetization structure of a moiré super cell of small-angle twisted bilayer CrI_3. a Lattice relaxation of two twisted CrI_3 monolayers. u_1 and u_2 describe local positions of a lattice site on layer 1 and layer 2, and Δu is the lattice relaxation induced spatial shift of u_2. **b** Schematic illustration of the relaxation vector Δu within a moiré cell of 0.25° twisted bilayer CrI_3. The size and direction of the arrows represent the magnitude and local direction of Δu. The moiré lattice structure relaxes to a periodic pattern consisting of monoclinic (M) and rhombohedral (R) stacking regions. **c** Theoretically calculated magnetization structure of a moiré cell of 0.25° twisted bilayer CrI_3. The central triangular area showing zero net magnetic moment represents the AFM state while the remaining areas with uncompensated net magnetization correspond to the FM phase.

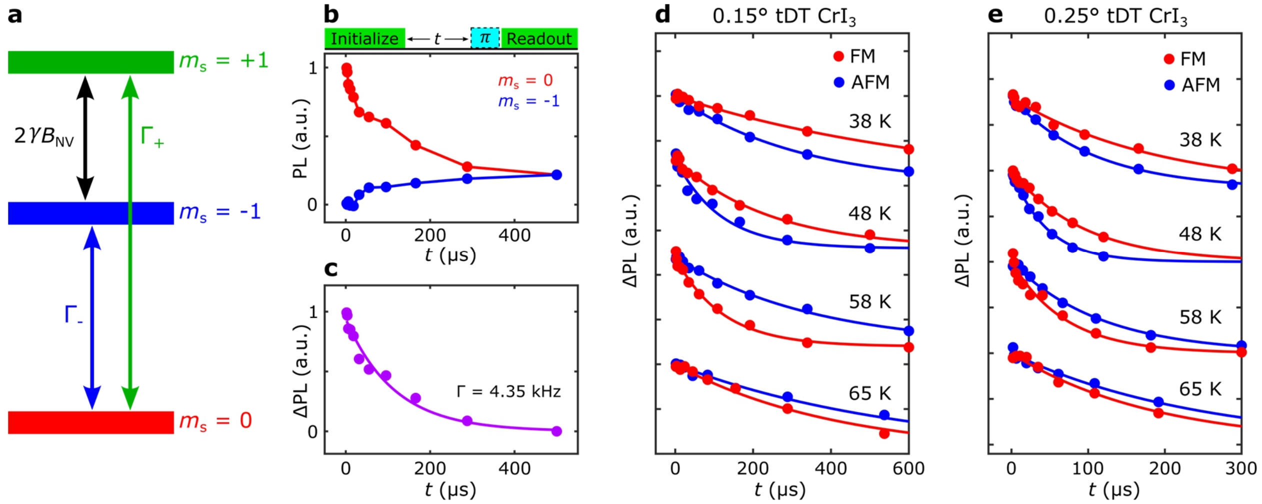

Supplementary Fig. 7. Extraction of NV spin relaxation rates. a NV spin relaxation in a three-level system. Γ_+ and Γ_- represent the NV spin relaxation rates of the $m_s = 0 \leftrightarrow +1$ and $m_s = 0 \leftrightarrow -1$ transition, respectively. **b** Top panel: optical and microwave measurement sequence for NV relaxometry measurements. Bottom panel: A typical set of NV relaxometry data measured on a tDT CrI_3 sample showing spin dependent NV PL curves of $m_s = 0$ and $m_s = -1$ states as a function of delay time *t*. **c** Difference (ΔPL) of the NV PL curves of the $m_s = 0$ and $m_s = -1$ states presented in **b**. NV relaxation rate Γ is obtained to be 4.35 kHz by fitting the ΔPL spectrum to Eq. (14) in Supplementary Information. **d-e** Sets of NV spin relaxometry data showing the ΔPL spectrum as a function of the delay time *t* measured on 0.15° tDT CrI_3 (**d**) and 0.25° tDT CrI_3 (**e**) device at different temperatures. Red and blue data points (curves) are taken when the NV spin sensor is positioned right above the FM and AFM regions in tDT CrI_3.

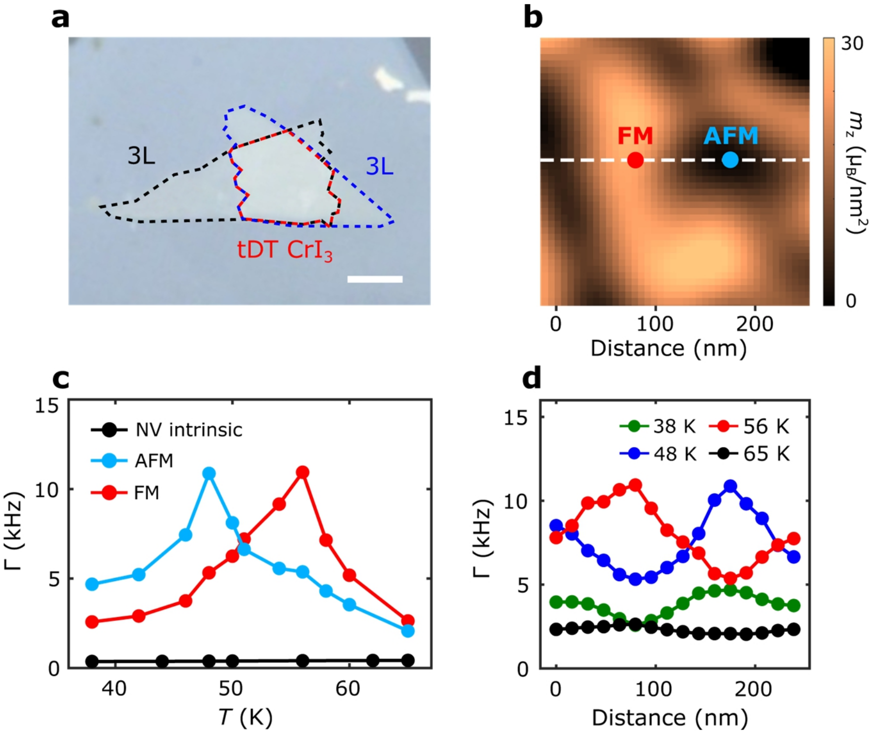

Supplementary Fig. 8. NV spin relaxometry measurements of 0.25° tDT CrI_3. a Optical microscope image of a prepared 0.25° tDT CrI_3 sample. The two torn trilayer CrI_3 flakes are outlined by the black and blue dashed lines, and the twisted area is highlighted by the red dashed lines. Scale bar is 5 μm. **b** Zoomed-in view of a magnetization map measured on a selected twisted sample area (250 nm × 250 nm) of the 0.25° tDT CrI_3 device, showing co-existing FM and AFM domains. Red and blue dots mark the central positions of the FM and AFM domains. The scale bar is 200 nm. **c** Temperature dependence of NV spin relaxation rate Γ measured when the NV center is positioned right above the FM and AFM domains formed in the 0.25° tDT CrI_3 sample. Control measurement results are also presented to characterize the intrinsic NV spin relaxation rate when the NV center is retracted away from sample surface. **d** 1D NV spin relaxation rate Γ measured along the white dashed lines across the FM and AFM domains in the 0.25° tDT CrI_3 sample at different temperatures. Note that the peak values of Γ at 48 K and 56 K occur at the corresponding in-plane lateral positions of the AFM and FM domains.

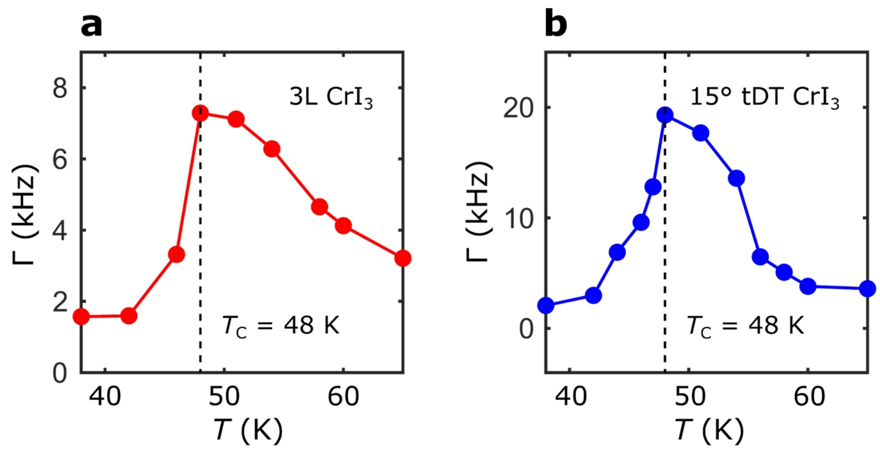

Supplementary Fig. 9. NV spin relaxometry measurements for trilayer CrI_3 and 15° tDT CrI_3. a-b Temperature dependence of NV spin relaxation rate for a pristine trilayer CrI_3 (**a**) and 15° tDT CrI_3 (**b**) device. NV ESR frequency is set to be 0.5 GHz in these measurements with an external magnetic field B_{ext} = 1,207 G applied along the NV spin direction. The Curie temperatures of trilayer CrI_3 and 15° tDT CrI_3 are obtained to be ~48 K as highlighted by the black dashed lines.

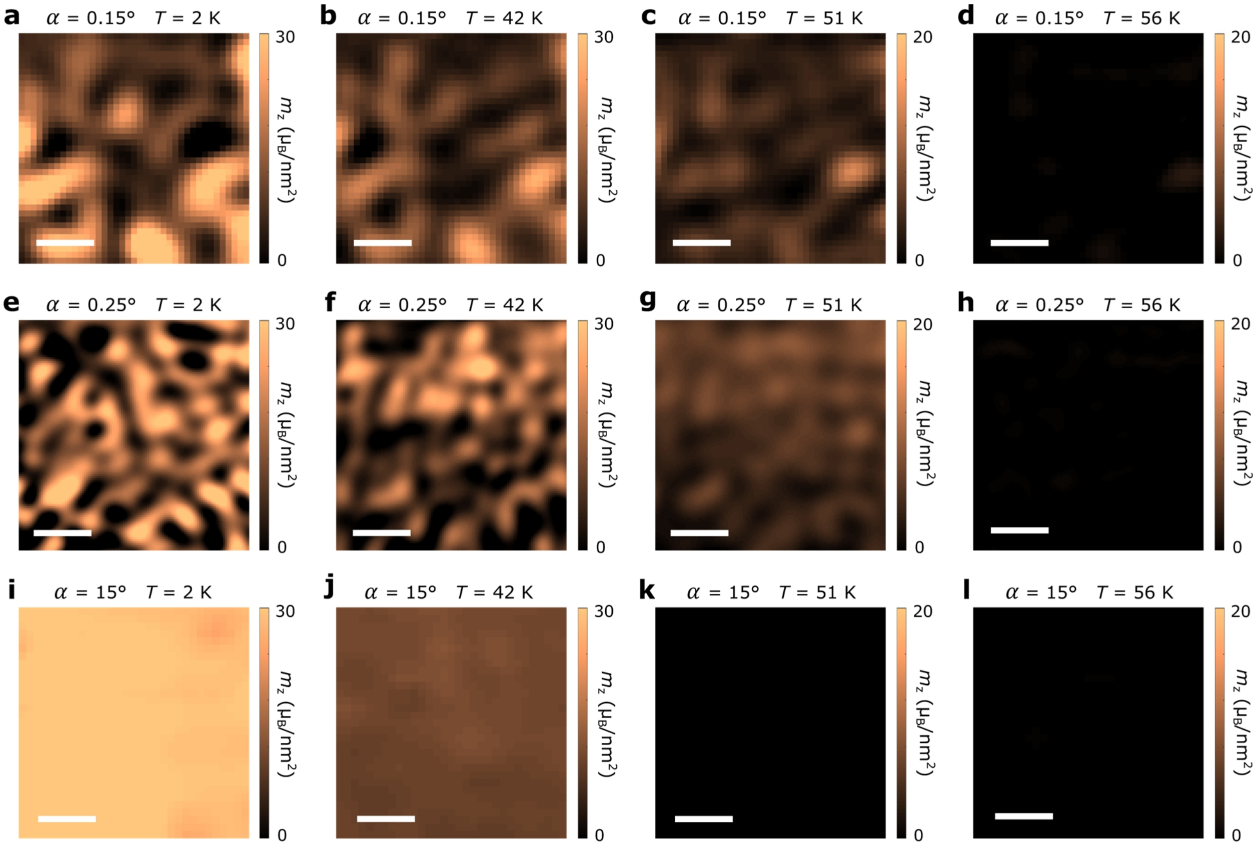

Supplementary Fig. 10. Extended scanning NV magnetization maps of tDT CrI_3 devices. a-l Reconstructed magnetization maps of surveyed sample areas of 0.15° tDT CrI_3, 0.25° tDT CrI_3, and 15° tDT CrI_3 devices at the corresponding temperatures. The scale bar is 200 nm.

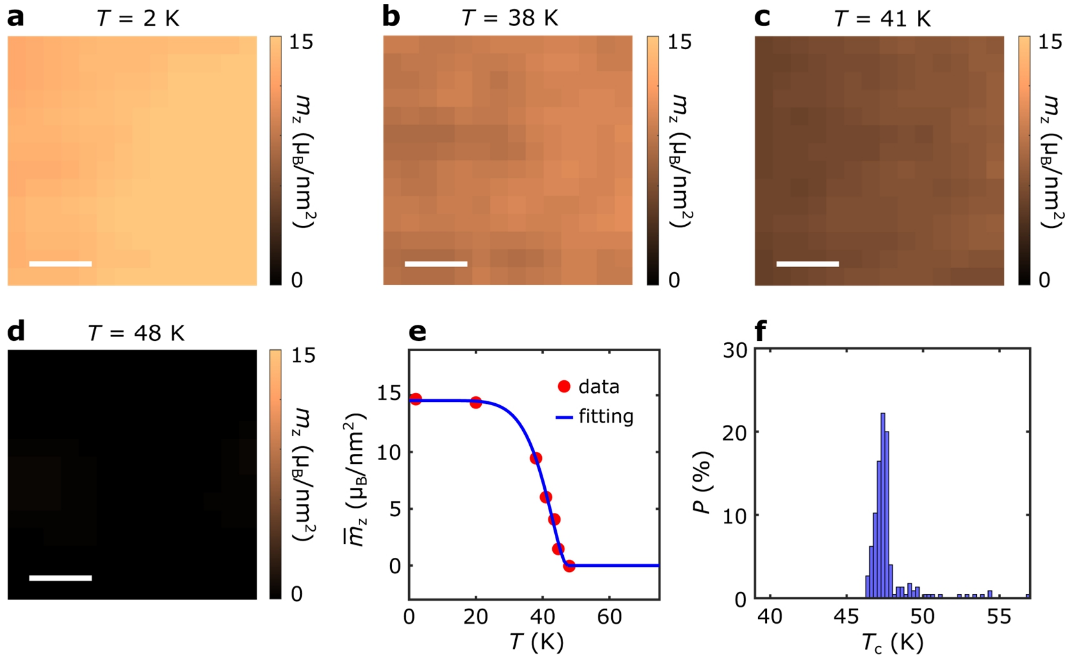

Supplementary Fig. 11. Scanning NV magnetometry measurements of trilayer CrI_3. a-d Reconstructed magnetization maps of a selected sample area of a pristine trilayer CrI_3 sample measured at 2 K (**a**), 38 K (**b**), 41 K (**c**), and 48 K (**d**). The scale bar is 100 nm. **e** Temperature dependence of the spatially averaged out-of-plane magnetization $\overline{m}_z$ in the probing area of trilayer CrI_3. The presented experimental results (red dots) are consistent with an empirical model (blue curve), from which the magnetic phase transition temperature is obtained to be ~48 K. **f** Histogram of obtained Curie temperatures T_c at individual pixels of the presented magnetization maps of trilayer CrI_3.
